# Zmpste24 deficiency contributes to intervertebral disc degeneration by undermining the stability of the nuclear membrane of nucleus pulposus cells

**DOI:** 10.7717/peerj.20534

**Published:** 2026-01-07

**Authors:** Chen Han, Shaotian Fu, Hanyi Wang, Kai Zhang, Jie Zhao

**Affiliations:** 1 Shanghai Jiao Tong University School of Medicine, Shanghai Ninth People’s Hospital, Shanghai, China; 2 Shanghai Jiao Tong University School of Medicine, Shanghai Jiao Tong University School of Medicine, Shanghai, China

**Keywords:** Zmpste24, Intervertebral disc degeneration, Nucleus pulposus cells, Nucleus, *Zmpste24* KO mice, Senescence, Mechanical stress, Nuclear membrane, Intervertebral disc, Inflammation

## Abstract

Intervertebral disc degeneration (IVDD) is often accompanied by the senescence of nucleus pulposus (NP) cells and narrowed intervertebral disc space. Zinc metalloproteinase STE24 (Zmpste24), a common anti-aging gene, has been studied in several diseases but remains understudied in IVDD. This study aimed to investigate the relationship between IVDD and alterations in Zmpste24 expression. Immunohistochemical staining revealed that reduced Zmpste24 expression in patients with IVDD. * In vitro* experiments using rat NP cells revealed that Zmpste24 inhibition induced nuclear instability and cellular senescence. In addition, the phenotype and immunohistochemical staining of * Zmpste24* knockout (KO) mice confirmed that Zmpste24 plays a protective role against IVDD. Collectively, these findings suggest that reduced Zmpste24 expression in NP cells may contribute to IVDD pathogenesis.

## Introduction

The intervertebral disc is composed of annulus fibrosus (AF), cartilaginous endplates, and nucleus pulposus (NP) cells. The intervertebral disc plays a critical role in the mechanical stability and movement of the vertebral column (DiStefano, Illien-Jünger & Iatridis, 2019; Iatridis et al., 2006; Zhao et al., 2007). Aging and incorrect mechanical load can lead to intervertebral disc degeneration (IVDD), a process characterized by senescence of NP cells and extracellular matrix (ECM) remodeling involving type II collagen degradation coupled with type I collagen hyperplasia (Sun et al., 2022). As hydration of the NP diminishes, its elasticity decreases, accompanied by disruption of the structural boundary between the NP and AF. The outer AF undergoes thinning, hyaline degeneration, and fissure formation, compromising its capacity to contain the NP (Feng et al., 2025). When the AF is subjected to trauma or mechanical stress, existing fissures may enlarge, allowing NP herniation through compromised regions (Li et al., 2025). This herniation can compress neural tissues, leading to pain, neurological dysfunction, and disability, significantly decreasing the quality of life of patients and increasing their socio-economic burden (Nixon, 1986).

A complex interplay of mechanical stress, inflammation, and neurological damage leads to low back pain among patients with IVDD (Coppes et al., 1990; Knezevic et al., 2021). Prolonged abnormal mechanical load breaks the AF and leads to the subsequent release of various inflammatory factors, such as TNF-*α*, IL-1, and matrix metalloproteinases (MMPs) by NP cells, which stimulate inflammatory cell aggregation and neuronal damage, thereby accelerating the progression of IVDD (Bonnevie et al., 2019). Zmpste24 is a heptameric transmembrane zinc metalloproteinase, which is expressed in various cells at a constant level. It is located in the inner nuclear membrane and organelles and preserves the integrity of the nucleus (Worman & Michaelis, 2023; Wu et al., 2014). Mutations of the ZMPSTE24 gene induce characteristic features of the Hutchinson-Gilford progeria syndrome, such as premature aging (Liu et al., 2005). Zmpste24 plays a prominent role in various senescence-related diseases. Existing studies have demonstrated that aging reduces Zmpste24 expression in osteoprogenitor cells, inducing cell apoptosis and decreasing ECM production and bone mass. However, mechanical stress changes associated with exercise can reverse Zmpste24 depletion (Yang et al., 2023). During aging-related osteoarthritis progression, reduced Zmpste24 expression in chondrocytes promotes presenilin accumulation, inhibits cell proliferation, and accelerates cellular senescence (Suo et al., 2023). Nevertheless, its role in the mechanosensing during disc pathology and the inflammatory response among patients with IVDD remains to be investigated.

This study aimed to investigate the role of Zmpste24 in the development of IVDD. Based on the analysis of clinical samples and mouse models, it was found that prolonged mechanical stress can decrease the expression of Zmpste24. *Zmpste24* KO mice exhibited a more severe IVDD phenotype, suggesting the importance of Zmpste24 in lumbar disc herniation. Thus, Zmpste24 may be a potential genetic marker and a therapeutic target for treating lumbar disc degeneration. In conclusion, our *in vivo* and *in vitro* experiments demonstrated that Zmpste24 plays a protective role against IVDD.

## Materials and Methods

### IVD tissue collection

Nine specimens of NP were obtained from nine patients undergoing spinal surgery in Shanghai Ninth People’s Hospital. According to the modified magnetic resonance imaging (MRI)-based Pfirrmann grading system (A four-category degenerative grading scale was established, with total scores ranging from 4 points (1 point per category) for normal discs to 12 points (3 points per category) for severely degenerated discs), they were classified into three groups: Pfirrmann III–IV, Pfirrmann V–VI, and Pfirrmann VII–VIII (Griffith et al., 2007). The study was approved by the Ethics Committee of Shanghai Ninth People’s Hospital (SH9H-2021-T94-2), and written informed consent was obtained from all participants before surgery.

### Animals

Five-week-old male Sprague-Dawley (SD) rats were used in NP cell isolation. *Zmpste24* KO male and female mice were raised in the animal resource center of Ninth People’s Hospital under specific-pathogen-free (SPF) conditions, ensure mice are free from infection or mortality, they can eat and drink freely in the SPF environment, 12-hour light/dark cycle (07: 00 to 19: 00 light), maintain the ambient temperature (22 °C)and 30–70% humidity, one to two toy equipment in the environment. The most suitable number of mice in each of the three groups should be three to four, so three mice were selected for each group. When the mice reached 8 weeks of age, through the computer, three mice were randomly selected from each cage of numbered mice with known genotypes, and then experiments were conducted on these selected mice. During the treatment and measurement, different mice in different cages were randomly selected for measurement. All mice had the same diet and living environment. X-ray imaging was performed under isoflurane anesthesia, followed by euthanasia, all experimental procedures were conducted in an SPF-grade environment. We have provided a detailed experimental plan and declaration to the Laboratory Animal Ethics Committee of Shanghai Ninth People’s Hospital when applying for animal ethics permits (SH9H-2021-A607-SB).

### Isolation and culture of primary NP cells

Five-week-old male SD rats were euthanized *via* intraperitoneal injection of pentobarbital (50 mg/kg) and sterilized in 75% ethanol for 15 min. Thereafter, the tail skin of rats was cut to expose the intervertebral disc, the cartilage endplate was resected, and the NP was digested in 1% collagenase type II at 37 °C for 15 min. After centrifugation and resuspension, cells were transferred to the DMEM medium containing 10% FBS and 1% penicillin-streptomycin (Basatvat et al., 2023). Cells were cultured at 37 °C in an incubator of 21% oxygen and 5% carbon dioxide. All experiments were performed using cells from passages 2–4 (P2-P4). The Sprague-Dawley rats utilized in this study were reared in compliance with ethical standards and are housed in compliance with SPF standards.

### *Zmpste24* knockout

Initially, the sgRNA expression vector targeting Zmpste24 was designed and cloned, with the sequence provided in the Table S1. Subsequently, Cas9 mRNA and sgRNA were transcribed *in vitro*. Following this, fertilized embryo microinjection and embryo transfer were conducted. The resultant mice were genotyped using PCR and sequencing techniques. Screening of the F1 generation was carried out, yielding 3–5 F1 knockout mice. The genetic background of all mice was C57BL/6J, with experimental groups consisting of *Zmpste24* KO or heterozygous (hets) mice. None of the mice had prior pharmacological interventions, surgical procedures, or vaccinations.

### qPCR

RNA extraction and analysis were conducted using quantitative real-time PCR (qPCR). To explore the effect of lopinavir (ABT-378) on *Zmpste24*, *LMNA*, *type II collagen (Col2a1)*, *matrix metalloproteinase 3 (MMP3)*, 3 × 10^5^ cells/well were cultured in DMEM on 6-well plates. 24 h later, the NP cells were treated with control medium or lopinavir (10 µM) for 24 h. After incubation, NP cells were incubated with the Axygen RNA minipreparation kit (Axygen, Union City, CA, USA) to extract total RNA. The RNA sample was then reverse-transcribed into cDNA using the Prime Script RT Master Mix Kit. SYBR Premix Ex Taq (Takara 420A, Shiga, Japan) was used to perform real-time qPCR, using the 2-ΔCT method, relative mRNA expression levels were calculated and normalized against *β*-actin expression. The primer sequences were designed using NCBI BLAST (Table S2).

### RNA extraction from mouse NP tissues

Eight-week-old male WT and *Zmpste24* KO mice were euthanized, followed by careful dissection of translucent and soft NP tissues from intervertebral discs using scissors and forceps. The isolated tissues were immediately placed on ice and thoroughly ground into fine powder using a pre-chilled mortar and pestle. Total RNA was extracted from the homogenized tissues according to previously established protocols. qPCR was performed to quantify mRNA expression levels of *p16* and *p21*. The primer sequences were designed using NCBI BLAST (Table S2).

### Histological analysis

Mice of different sexes were euthanized at the end of the experiment, and the spine was isolated. The specimens were first fixed in 4% paraformaldehyde for 24 h, and then soaked in 75% ethanol for histological and radiological assessments. After stirring for 48 h in 4% paraformaldehyde, the stirred spines were decalcified in 10% EDTA (pH 7.4) for 21 days. Then, each tissue was dehydrated with different concentrations of ethanol, cleared with xylene, and paraffin-embedded. Next, hematoxylin and eosin (H&E) staining and safranin O/fast green were performed. Histological scores of mouse intervertebral discs were obtained according to a previously described classification system, which evaluated the cellular composition and morphological characteristics of the AF, NP, and their interface. The scoring criteria encompassed five degenerative categories, with total scores ranging from 0 points (normal disc, 0 points per category) to 15 points (severely degenerated disc, 3 points per category) (Ji et al., 2018).

### Immunofluorescence

Cells were rinsed three times with PBS and fixed with 4% paraformaldehyde for 15 min at room temperature, followed by permeabilization with 0.1% Triton X-100 in PBS for 30 min. After blocking non-specific binding sites with 3% BSA/PBS for 1 h, cells were incubated overnight at 4 °C with rabbit monoclonal primary antibodies diluted in 1% BSA/PBS: anti-Zmpste24 (1:200, #13426-1-AP; Proteintech, Rosemont, IL, USA) or anti-Lamin A (1:200, #12592-1-AP; Proteintech, Rosemont, IL, USA). The following day, cells were incubated with Alexa Fluor 555-conjugated goat anti-rabbit IgG secondary antibody (1:300, Abcam, Cambridge, UK) for 1 h at room temperature in darkness. For cytoskeletal visualization, F-actin was stained using phalloidin-iFluor 647 (1:500, Abcam) for 1 h, followed by nuclear counterstaining with DAPI (Beyotime, Shanghai, China) for 15 min. Confocal imaging was performed using a Zeiss LSM 880 laser scanning confocal microscope equipped with Airyscan super-resolution technology.

### Immunohistochemistry

For immunohistochemical staining, mouse disc samples were incubated with primary antibodies against COL2A1 (1:400, #ab34712; Abcam, Cambridge, UK) and AGGRECAN (1:2000, #ab313636; Abcam, Cambridge, UK), followed by incubation with the corresponding secondary antibody, anti-rabbit IgG [H+L] (1:1000, CST, #4412). The sections were incubated with DAPI in the dark for 10 min. Finally, all sections were subjected to a final wash with PBS, air-dried, and sealed with an anti-fluorescence tablet. Images were captured using an epifluorescence microscope (Leica Microsystems), and integrated Optical Density (IOD) was analyzed using Image J software.

### Senescence-associated *β*-galactosidase staining

NP progenitor cells were seeded onto 6-well plates at a density of 3 × 10^5^ cells per well and treated with lopinavir (10 µM) for 24 h. After washing with PBS once, the chondrocytes were fixed in 4% paraformaldehyde for 15 min and stained with senescence-associated *β*-galactosidase (SA-*β*-gal) staining kit (Beyotime, China). Images were taken using a Zeiss microscope, and integrated Optical Density (IOD) was analyzed using Image J software.

### High-density culture

In high-density culture, approximately 150,000 NP progenitor cells were re-suspended in 10 µL control medium and seeded as NP cells clusters in 12-well plates to study the ECM secretion capacity of NP cells. NP progenitor cells were allowed to adhere to the bottom at 37 °C for 1 h. Then, 1 ml of MEM/F12 medium containing 10 ng/ml ITS (insulin-transferrin-selenium; Cyagen, Santa Clara, CA, USA) and 2% FBS was added. Lopinavir (10 µM) was included in the experimental groups. The medium was replaced every 2 days, and after 9 days, these NP cells clusters were stained with Alcian blue.

### X-ray

Eight-week-old male and female *Zmpste24* KO and WT mice were divided into four groups, with three mice per group (totaling 12 mice). All groups were housed in individual cages. They were subjected to X-ray imaging to assess their intervertebral disc alterations. The digital images were captured in the anteroposterior projection using a 21 lp/mm detector, which provides up to 5× geometric magnification (Faxitron VersaVision; Faxitron Bioptics LLC, Tucson, AZ, USA).

### Micro-CT

Following X-ray imaging, the aforementioned 12 mice were euthanized, and their spinal columns were excised for subsequent micro-CT analysis. A high-resolution micro-CT (µCT-100, SCANCO Medical AG, Switzerland) was used to perform microcomputed tomography (micro-CT). The scans were immersed in 75% ethanol using an X-ray intensity of 200 µA, an X-ray tube voltage of 70 kVp, and an integration time of 300 ms. We analyzed the three-dimensional shape of the region of interest (ROI) to assess vertebral bone size and intervertebral disc space. using a previously validated formula, the disc height indexes (DHI) were calculated (Han et al., 2008).

### Statistical analysis

A predefined experimental timeline was followed, with all data recorded rigorously. Blended methodology was applied during both data collection and statistical analysis, with all data being faithfully analyzed. Three biological replicates were performed. Data are presented as the mean ± standard deviation. Statistical comparisons were conducted using GraphPad Prism 7.0 (GraphPad Software, San Diego, CA, United States). Student’s *t*-test was used to assess differences between two groups. one-way ANOVA for comparisons among multiple groups. Statistical significance was defined as *p* < 0.05.

## Results

### The expression of ZMPSTE24 in NP cells decreased after IVDD

**Figure 1:**
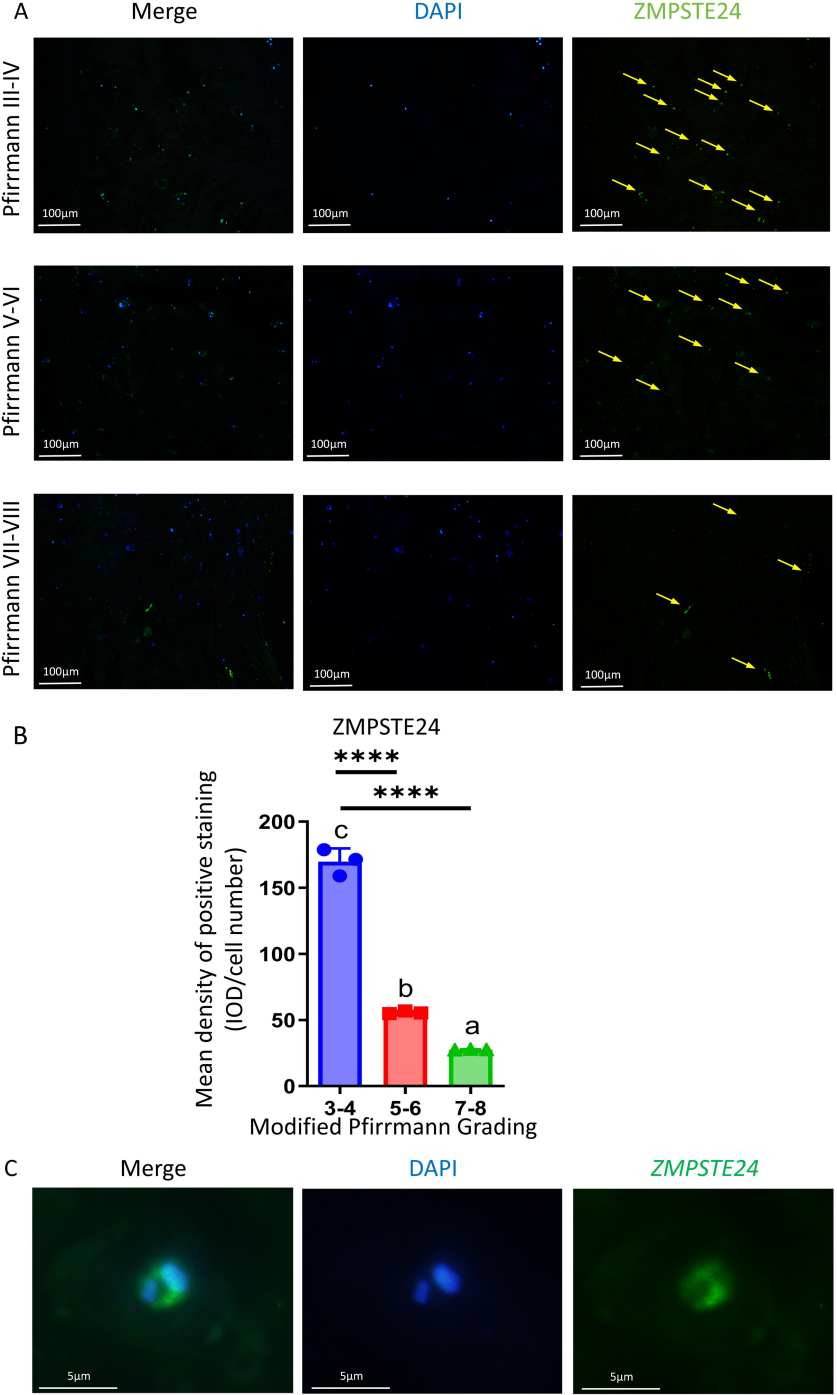
Decreased ZMPSTE24 was found in NP samples from patients with intervertebral disc degeneration. (A) Comparison of IF staining of ZMPSTE24 in the NP of Pfirrmann grading III–IV, Pfirrmann grading V-VI and Pfirrmann grading VII–VIII patients. (B) Mean density of positive staining of ZMPSTE24 in NP cells from patients with different Pfirrmann Grading classifications (*n* = 3) ^∗^*p* < 0.05, ^∗∗^*p* < 0.01, ^∗∗∗^*p* < 0.001, and ^∗∗∗∗^*p* < 0.0001. (C) Localization of ZMPSTE24 in human NP cells.

Mice with *Zmpste24* KO exhibit premature aging phenotypes in previous studies (Liu et al., 2005). To investigate ZMPSTE24 expression in human IVDD, we collected NP tissues from patients, classified according to the modified Pfirrmann grading system (Masuda et al., 2005), and conducted immunofluorescence analysis after culturing. Our findings revealed detectable ZMPSTE24 expression in NP tissues from IVDD patients (Fig. 1A). Notably, ZMPSTE24 levels were significantly downregulated in tissues with high Pfirrmann scores (grades IV–V), suggesting severe disc degeneration (Fig. 1B). Fluorescence microscopy revealed that ZMPSTE24 localizes to the nuclear periphery of NP cells, and ZMPSTE24 was distributed around the nucleus of NP cells (Fig. 1C). This localization pattern aligns with its established role in maintaining nuclear envelope integrity and mediating mechanotransduction. Therefore, decreased Zmpste24 expression suggests compromised nuclear membrane stability in NP cells.

### Inhibition of Zmpste24 led to NP cell senescence and undermined their ability to secrete ECM

**Figure 2:**
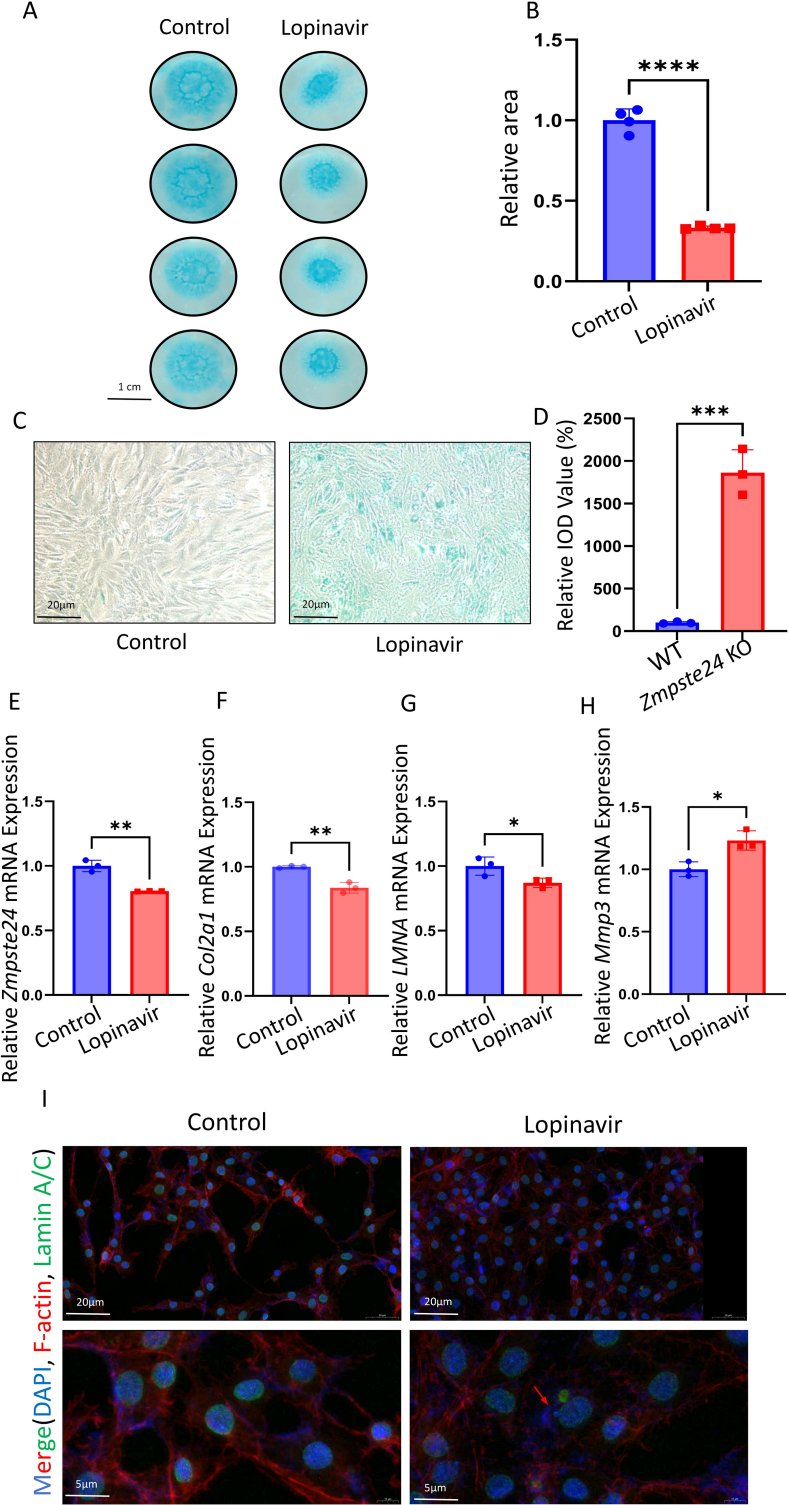
Inhibition of Zmpste24 in rat NP cells resulted in cell senescence. (A–B) Comparison of high-density culture area of rat NP cells between control group (*n* = 4) and Lopinavir group (*n* = 4) ^∗^*p* < 0.05, ^∗∗^*p* < 0.01, ^∗∗∗^*p* < 0.001, and ^∗∗∗∗^*p* < 0.0001. (C) *β* -gal staining experiment for detecting the degree of NP cells senescence. (D) Statistics of relative IOD value (%) of *β*-gal staining experiment respectively (*n* = 3) ^∗^*p* < 0.05, ^∗∗^*p* < 0.01, ^∗∗∗^*p* < 0.001, and ^∗∗∗∗^*p* < 0.0001. (E–H) The relative mRNA expression levels of *Zmpste24*, *Col2a1*, *LMNA* and *Mmp3* in rat NP cells after lopinavir treatment (*n* = 3) ^∗^*p* < 0.05, ^∗∗^*p* < 0.01, ^∗∗∗^*p* < 0.001 and ^∗∗∗∗^*p* < 0.0001. (I) F-actin stained cell morphology and Lamin A/C immunofluorescence of NP cells from control and lopinavir induced senescence groups.

Rat NP cells were isolated and conducted an *in vitro* high-density assay (Fig. 2A). Cells were treated with lopinavir (10 µM, Zmpste24 inhibitor). Compared with untreated NP cells, NP cells treated with lopinavir exhibited reduced Alcian blue staining intensity and diminished staining area (Fig. 2B). Consistently, *β*-galactosidase staining revealed that lopinavir induced premature cellular senescence in NP cells, with a nearly 20-fold increase in integrated optical density (IOD) compared to untreated groups (Figs. 2C, 2D). qPCR revealed that rat NP cells stimulated with lopinavir had decreased *Zmpste24* expression (Fig. 2E) and reduced mRNA levels of *LMNA* (Fig. 2G). The expression and maturation of Lamin A/C depend on the Zmpste24 metalloproteinase. Furthermore, decreased *Col2a1* expression and increased *Mmp3* expression suggested that lopinavir promoted ECM degradation (Figs. 2F and 2H). In addition, lopinavir induced the senescence in NP cells. We also verified our findings with immunofluorescence in rat NP cells. Lopinavir led to the nuclear translocation of Lamin A with punctate distribution (Fig. 2I), suggesting that Zmpste24 inhibition by lopinavir affects the expression and distribution of Lamin A, destabilizes nuclear architecture, and promotes cellular senescence.

### *Zmpste24* KO mice showed a marked aging phenotype with decreased intervertebral space and body length

**Figure 3:**
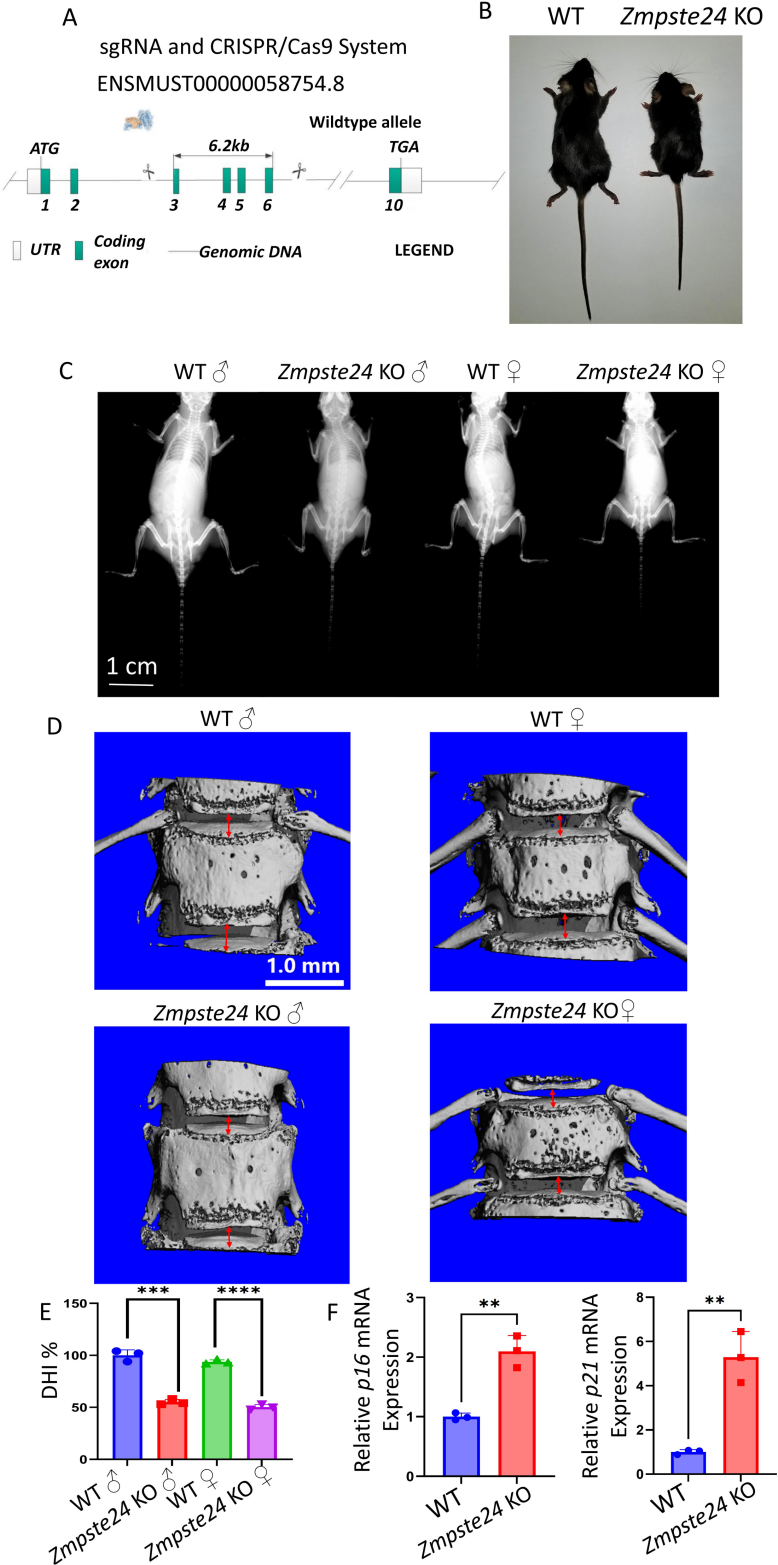
Zmpste24 KO mice showed aging phenotypes such as decreased intervertebral space height and body shortening. (A) Construction strategy of Zmpste24 KO mice. (B) Comparison of body size between WT and Zmpste24 KO mice. (C) Comparison of X-ray films of male WT mice, male Zmpste24 KO mice, female WT mice and female Zmpste24 KO mice (*n* = 3). (D) Comparison of intervertebral space height under CT reconstruction in male WT mice, male Zmpste24 KO mice, female WT mice and female Zmpste24 KO mice (*n* = 3). (E) Statistical plot of DHI% for the four types of mice (*n* = 3) ^∗^*p* < 0.05, ^∗∗^*p* < 0.01, ^∗∗∗^*p* < 0.001, and ^∗∗∗∗^*p* < 0.0001. (F) The relative mRNA expression levels of p16 and p2 1 in the NP tissue of WT and Zmpste24 KO mice (*n* = 3) ^∗^*p* < 0.05, ^∗∗^*p* < 0.01, ^∗∗∗^*p* < 0.001 and ^∗∗∗∗^*p* < 0.0001.

We generated *Zmpste24* KO mice *via* CRISPR/Cas9-mediated gene editing (Fig. 3A). The KO mice exhibited a significant decrease in body size compared to wild-type (WT) mice of the same age (Fig. 3B). X-ray also showed shorter body lengths and limbs among KO mice compared to WT mice of the same age and sex (Fig. 3C). Subsequently, we performed high-resolution micro-CT analysis on male and female KO mice and WT mice of the same age and sex to quantify vertebral body height and intervertebral disc height (Figs. 3D and 3E). Sex-stratified analysis demonstrated that male KO mice exhibited approximately 50% reduction in intervertebral disc height compared to age-matched male WT controls. Similarly, female KO mice showed significantly diminished disc height relative to female WT counterparts, confirming consistent phenotypic manifestation across sexes. Quantitative RT-PCR analysis of NP tissues revealed marked upregulation of senescence-associated genes in KO mice, with *p16* and *p21* mRNA levels increased 2-fold and 5-fold respectively, mirroring the premature senescence phenotype observed in lopinavir-treated NP cells. These findings collectively indicate that *Zmpste24* deficiency accelerates age-related spinal degeneration.

### Significant disc height reduction, NP shrinkage, and AF thickness were observed in *Zmpste24* KO and *Zmpste24* hets mice

**Figure 4:**
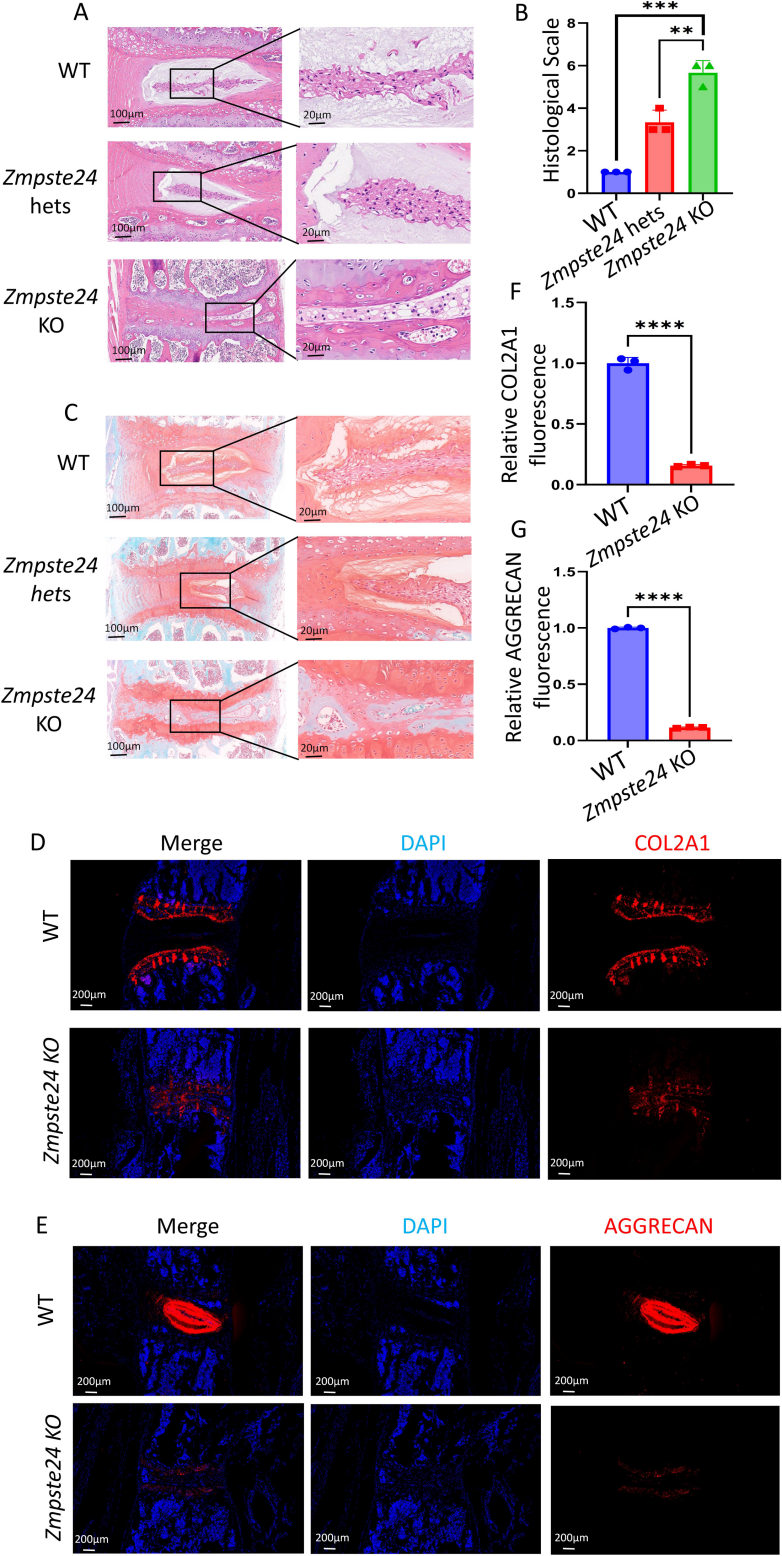
Progressive worsening of the spine aging phenotype between WT, *Zmpste24* hets and *Zmpste24* KO mice. *Zmpste24* KO mice show decreased expression of proteins involved in soft tissue development and bone development, leading to a spine-aging phenotype. (A) HE staining of spinal discs from WT, *Zmpste24* hets and *Zmpste24* KO mice. (B) Statistical plots of histological scale in mice of the three genotypes (*n* = 3) ^∗^*p* < 0.05, ^∗∗^*p* < 0.01, ^∗∗∗^*p* < 0.001, and ^∗∗∗∗^*p* < 0.0001. (C) Safranin-fast green staining of spinal discs from WT, *Zmpste24* hets, and *Zmpste24* KO mice. (D, F) Comparison of IF staining of COL2A1 in the spine of WT and *Zmpste24* KO mice and statistical plot of the relative fluorescence intensity of COL2A1 (*n* = 3). (E, G) Comparison of IF staining of AGGRECAN in the spine of WT and *Zmpste24* KO mice and statistical plot of the relative fluorescence intensity of AGGRECAN (*n* = 3).

H&E staining revealed narrowed intervertebral disc space among *Zmpste24* KO mice compared to WT mice (Fig. 4A). Additionally, the AF of the intervertebral disc was sparse in *Zmpste24* KO mice, with significant atrophy and reduced size of NP (Fig. 4B). These findings correlated with reduced body length in *Zmpste24* KO mice compared to WT mice. The disc size and body length of hets mice were between those of KO and WT groups, indicating graded spinal degeneration. These findings suggest that *Zmpste24*, as an anti-aging gene, plays a crucial role in normal development and degeneration of the intervertebral disc, in addition to maintaining cell membrane stability in the NP. Safranin O/green staining revealed a significant decrease in the size of the NP and thickness of the AF in *Zmpste24* KO mice. *Zmpste24* KO mice also exhibited decreased ECM content (Fig. 4C). In contrast, the senescence phenotype of hets mice was between that of WT and KO mice, consistent with H&E findings.

### The aging phenotype of *Zmpste24* KO mice was associated with decreased expression of COL2A1 and AGGRECAN

Immunofluorescence showed differences in the expression of intervertebral disc-related ECM proteins between KO and WT groups. COL2A1 and AGGRECAN, critical components of endplate cartilage and NP ECM, exhibited markedly reduced expression in aging-related pathologies. Given the hypocellular and hydrated nature of NP tissue, immunostaining for COL2A1 showed clearer visualization in the cartilaginous endplates. Quantitative analysis demonstrated that *Zmpste24* KO mice exhibited significantly diminished COL2A1 expression in intervertebral discs compared to WT controls (Figs. 4D, 4F), accompanied by reduced distance between superior and inferior cartilaginous endplates. Similarly, AGGRECAN immunoreactivity was substantially lower in the NP of KO mice relative to age-matched WT littermates (Figs. 4E, 4G). This finding indicates that *Zmpste24* KO disrupts ECM protein synthesis, compromising structural integrity, impairing the development of vertebrae and intervertebral discs.

## Discussion

As a key enzyme in the conversion of precursor prelamin A to mature Lamin A, ZMPSTE24 plays a critical role in human health (Quigley et al., 2013). Abnormal expression of Zmpste24 can induce premature senescence, Hutchinson-Gilford Progeria syndrome, mandibuloacral dysplasia-type B, and restrictive dermopathy (Eriksson et al., 2003; Kwan, 2015; Odinammadu et al., 2023). In addition to classic premature aging syndromes, Zmpste24 has recently been found to play an important role in several age-related diseases, such as osteoarthritis, osteoporosis, and atherosclerosis (Ragnauth et al., 2010). Despite these associations, the functional involvement of Zmpste24 in IVDD remains underexplored. Our study was the first to systematically investigate Zmpste24-IVDD relationships. We initially isolated NP cells from patients with IVDD and observed a significant inverse correlation between Zmpste24 downregulation and the severity of disc degeneration. *In vitro* experiments using rat NP cells demonstrated that pharmacological inhibition of Zmpste24 with lopinavir markedly suppressed ECM synthesis, promoted cellular senescence, and disrupted nuclear envelope integrity, which consistent with the Zmpste24’s established cellular functions. To validate these observations, we generated *Zmpste24* KO mice. In the intervertebral disc tissues of *Zmpste24* KO mice, the expression levels of senescence-associated genes *p16* and *p21* were significantly elevated, while the synthesis of ECM components, exemplified by COL2A1 and AGGRECAN, was substantially diminished. Collectively, *Zmpste24* KO significantly decreased intervertebral space height, induced the shrinkage of the NP, and AF degeneration in mice. These findings establish a promising opportunity for further research into intervertebral disc aging.

Rodents retain a high proportion of notochordal cells in adult intervertebral discs, which confer natural regenerative and anti-degenerative properties to the NP, yet mice and rats remain predominant in IVDD research due to their genetic manipulability for mechanistic studies, tractability in investigating cellular senescence under stressors like stemness loss and cell cycle arrest, and phenotypic parallels with human disc degeneration including nucleus desiccation, AF thinning, and disc height reduction (Poletto et al., 2023; Singh, Masuda & An, 2005; Tripathi, Bhombe & Kumar, 2025). While cynomolgus monkeys and goats better replicate human biomechanics and disc dimensions, their practical limitations such as high costs and genetic intractability restrict widespread use (Chen et al., 2022; Tong et al., 2017). As an initial research step, we will establish a clinical cohort of IVDD patients to monitor Zmpste24 expression changes in NP before and after treatment, complemented by higher-tier animal studies to validate translational relevance.

NP cells are specialized chondrocyte-like cells that secrete ECM components crucial for disc structure and shock absorption (Kamatani et al., 2022). Previous studies showed that Zmpste24 inhibits chondrocyte senescence and reduces osteoarthritis (Kong et al., 2025). Our experiments confirmed this: blocking or deleting Zmpste24 accelerates NP cell senescence and lumbar disc degeneration. However, our study had some limitations. First, although we conducted *in vitro* validation, we did not identify the molecular mechanisms and related pathways. Second, we observed decreased Zmpste24 expression and premature intervertebral disc senescence in aged animal models. However, we did not generate Zmpste24 overexpression to validate its protective effects. Third, despite observing elevated *p16* and *p21* in the NP tissue of conventional *Zmpste24* KO mice, we cannot preclude contributions from other disc-resident cells, such as osteocytes, fibroblasts, or macrophages. Thus, we plan to generate NP cell-specific conditional *Zmpste24* KO mice to confirm the disc degeneration phenotype. Then we will establish the overexpression model in both HEK-293T cells and NP cells and investigate the underlying molecular mechanisms and specific protective effects.

Recently, numerous innovative therapeutic approaches have emerged for treating IVDD (Frapin et al., 2019; Yang et al., 2024; Zhu et al., 2015), including disc-targeted drug delivery systems, anti-decay and anti-apoptosis therapeutics for intervertebral disc cells, stem cells and fibroblasts for IVDD, and exosomes and small RNAs. These approaches promote the regeneration and repair of the intervertebral disc (Barcellona et al., 2021; Binch et al., 2021; Fiordalisi et al., 2020). Zmpste24, an anti-aging protein, plays a crucial role in maintaining homeostasis in infectious and inflammatory diseases and natural aging (Kong et al., 2023; Messner et al., 2020). It is involved in the homeostasis of mitochondria, nuclei, and other organelles (Sieprath et al., 2015). However, it remains unknown whether any of these cutting-edge therapies enhance Zmpste24 function or expression in NP cells. Our study will investigate the specific mechanisms linking intervertebral disc aging to Zmpste24, with the aim of advancing gene therapy research for IVDD.

## Conclusion

**Figure 5:**
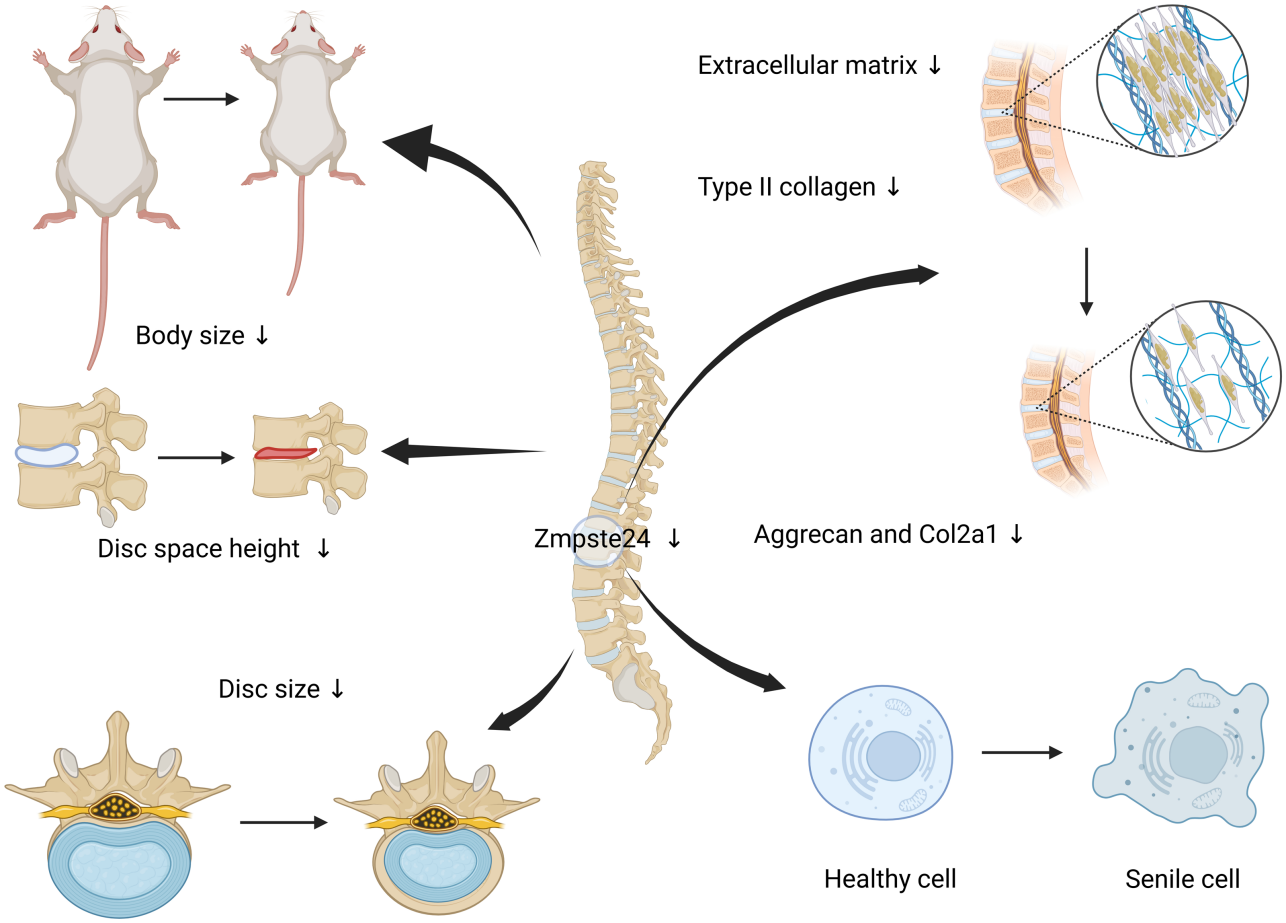
Diminished Zmpste24 expression in NP cells triggers cellular senescence and accelerates IVDD. Reduced Zmpste24 expression in NP cells induces premature cellular senescence, diminishes extracellular matrix secretion, and ultimately results in shortened body length and decreased intervertebral disc height in mice. Created in BioRender by Yang Xiao https://BioRender.com/uk58gn0.

In summary, for the first time, we found that Zmpste24 deficiency induces the symptoms of IVDD, including decreased disc height, shrinkage of the NP, and thickness of the AF. These findings were validated using clinical samples, *in vitro* experiments, and *Zmpste24* KO mice. Zmpste24 functions as a critical regulator of nuclear Lamin A processing and maintains nuclear stability (Fong et al., 2006; Fong et al., 2004). Intervertebral disc herniation is associated with the senescence of the NP and AF (Song et al., 2023). Our findings suggest the protective role of Zmpste24 in IVDD and provide a foundation for developing new therapies of patients with disc degeneration (Fig. 5).

##  Supplemental Information

10.7717/peerj.20534/supp-1 Supplemental Information 1 Raw data of Figs. 1 and 2

10.7717/peerj.20534/supp-2 Supplemental Information 2 Raw data of Fig. 3

10.7717/peerj.20534/supp-3 Supplemental Information 3 Raw data of Fig. 4A

10.7717/peerj.20534/supp-4 Supplemental Information 4 Raw data of Fig. 4B

10.7717/peerj.20534/supp-5 Supplemental Information 5 Dataset 1

10.7717/peerj.20534/supp-6 Supplemental Information 6 Dataset 2

10.7717/peerj.20534/supp-7 Supplemental Information 7 Supplementary tables

10.7717/peerj.20534/supp-8 Supplemental Information 8 MIQE checklist

10.7717/peerj.20534/supp-9 Supplemental Information 9 ARRIVE checklist

## Additional Information and Declarations

### Data Availability

The following information was supplied regarding data availability: All raw data supporting the findings of this study are available in the Supplemental Files.

## References

[ref-1] Barcellona MN, Speer JE, Jing L, Patil DS, Gupta MC, Buchowski JM, Setton LA (2021). Bioactive in situ crosslinkable polymer-peptide hydrogel for cell delivery to the intervertebral disc in a rat model. Acta Biomaterialia.

[ref-2] Basatvat S, Bach FC, Barcellona MN, Binch AL, Buckley CT, Bueno B, Chahine NO, Chee A, Creemers LB, Dudli S, Fearing B, Ferguson SJ, Gansau J, Gantenbein B, Gawri R, Glaeser JD, Grad S, Guerrero J, Haglund L, Hernandez PA, Hoyland JA, Huang C, Iatridis JC, Illien-Junger S, Jing L, Kraus P, Laagland LT, Lang G, Leung V, Li Z, Lufkin T, van Maanen JC, McDonnell EE, Panebianco CJ, Presciutti SM, Rao S, Richardson SM, Romereim S, Schmitz TC, Schol J, Setton L, Sheyn D, Snuggs JW, Sun Y, Tan X, Tryfonidou MA, Vo N, Wang D, Williams B, Williams R, Yoon ST, Le Maitre CL (2023). Harmonization and standardization of nucleus pulposus cell extraction and culture methods. JOR Spine.

[ref-3] Binch ALA, Fitzgerald JC, Growney EA, Barry F (2021). Cell-based strategies for IVD repair: clinical progress and translational obstacles. Nature Reviews Rheumatology.

[ref-4] Bonnevie ED, Gullbrand SE, Ashinsky BG, Tsinman TK, Elliott DM, Chao PG, Smith HE, Mauck RL (2019). Aberrant mechanosensing in injured intervertebral discs as a result of boundary-constraint disruption and residual-strain loss. Nature Biomedical Engineering.

[ref-5] Chen X, Chen H, Li BL, Xiao Z, Zhou Y, Tian W, Chen D, Liu X, Zhou Z, Liu S (2022). Dynamic elastic modulus assessment of the early degeneration model of an intervertebral disc in cynomolgus monkeys with one strike loading. Computer Methods and Programs in Biomedicine.

[ref-6] Coppes MH, Marani E, Thomeer RT, Oudega M, Groen GJ (1990). Innervation of annulus fibrosis in low back pain. The Lancet.

[ref-7] Di Stefano TJ, Illien-Jünger S, Iatridis JC (2019). Homeostasis disrupted by strain mechanosensing. Nature Biomedical Engineering.

[ref-8] Eriksson M, Brown WT, Gordon LB, Glynn MW, Singer J, Scott L, Erdos MR, Robbins CM, Moses TY, Berglund P, Dutra A, Pak E, Durkin S, Csoka AB, Boehnke M, Glover TW, Collins FS (2003). Recurrent de novo point mutations in lamin A cause Hutchinson-Gilford progeria syndrome. Nature.

[ref-9] Feng C, Hu Z, Zhao M, Leng C, Li G, Yang F, Fan X (2025). Region-specific mitophagy in nucleus pulposus, annulus fibrosus, and cartilage endplate of intervertebral disc degeneration: mechanisms and therapeutic strategies. Frontiers in Pharmacology.

[ref-10] Fiordalisi M, Silva AJ, Barbosa M, Gonçalves R, Caldeira J (2020). Decellularized scaffolds for intervertebral disc regeneration. Trends in Biotechnology.

[ref-11] Fong LG, Ng JK, Lammerding J, Vickers TA, Meta M, Coté N, Gavino B, Qiao X, Chang SY, Young SR, Yang SH, Stewart CL, Lee RT, Bennett CF, Bergo MO, Young SG (2006). Prelamin A and lamin A appear to be dispensable in the nuclear lamina. Journal of Clinical Investigation.

[ref-12] Fong LG, Ng JK, Meta M, Coté N, Yang SH, Stewart CL, Sullivan T, Burghardt A, Majumdar S, Reue K, Bergo MO, Young SG (2004). Heterozygosity for Lmna deficiency eliminates the progeria-like phenotypes in Zmpste24-deficient mice. Proceedings of the National Academy of Sciences of the United States of America.

[ref-13] Frapin L, Clouet J, Delplace V, Fusellier M, Guicheux J, Le Visage C (2019). Lessons learned from intervertebral disc pathophysiology to guide rational design of sequential delivery systems for therapeutic biological factors. Advanced Drug Delivery Reviews.

[ref-14] Griffith JF, Wang YX, Antonio GE, Choi KC, Yu A, Ahuja AT, Leung PC (2007). Modified Pfirrmann grading system for lumbar intervertebral disc degeneration. Spine.

[ref-15] Han B, Zhu K, Li FC, Xiao YX, Feng J, Shi ZL, Lin M, Wang J, Chen QX (2008). A simple disc degeneration model induced by percutaneous needle puncture in the rat tail. Spine.

[ref-16] Iatridis JC, MacLean JJ, Roughley PJ, Alini M (2006). Effects of mechanical loading on intervertebral disc metabolism in vivo. The Journal of Bone and Joint Surgery.

[ref-17] Ji ML, Jiang H, Zhang XJ, Shi PL, Li C, Wu H, Wu XT, Wang YT, Wang C, Lu J (2018). Preclinical development of a microRNA-based therapy for intervertebral disc degeneration. Nature Communications.

[ref-18] Kamatani T, Hagizawa H, Yarimitsu S, Morioka M, Koyamatsu S, Sugimoto M, Kodama J, Yamane J, Ishiguro H, Shichino S, Abe K, Fujibuchi W, Fujie H, Kaito T, Tsumaki N (2022). Human iPS cell-derived cartilaginous tissue spatially and functionally replaces nucleus pulposus. Biomaterials.

[ref-19] Knezevic NN, Candido KD, Vlaeyen JWS, Van Zundert J, Cohen SP (2021). Low back pain. The Lancet.

[ref-20] Kong K, Jin M, Zhao C, Qiao H, Chen X, Li B, Rong K, Zhang P, Shan Y, Xu Z, Chang Y, Li H, Zhai Z (2023). Mechanical overloading leads to chondrocyte degeneration and senescence via Zmpste24-mediated nuclear membrane instability. iScience.

[ref-21] Kong K, Liu L, Zhang R, Chang Y, Shao Y, Zhao C, Qiao H, Jin M, Chen X, Shi W, Wu X, Fan W, Hu Y, Rong K, Zhang P, Li B, Zhang J, Ma P, Zhang X, Li H, Zhai Z (2025). AIDS patients suffer higher risk of advanced knee osteoarthritis progression due to lopinavir-induced Zmpste24 inhibition. Bone Research.

[ref-22] Kwan JM (2015). Mandibuloacral dysplasia type B in an infant: a rare progeroid genodermatosis. JAMA Dermatology.

[ref-23] Li S, Jiang W, Chen F, Qian J, Yang J (2025). The critical role of TRIM protein family in intervertebral disc degeneration: mechanistic insights and therapeutic perspectives. Frontiers in Cell and Developmental Biology.

[ref-24] Liu B, Wang J, Chan KM, Tjia WM, Deng W, Guan X, Huang JD, Li KM, Chau PY, Chen DJ, Pei D, Pendas AM, Cadiñanos J, López-Otín C, Tse HF, Hutchison C, Chen J, Cao Y, Cheah KS, Tryggvason K, Zhou Z (2005). Genomic instability in laminopathy-based premature aging. Nature Medicine.

[ref-25] Masuda K, Aota Y, Muehleman C, Imai Y, Okuma M, Thonar EJ, Andersson GB, An HS (2005). A novel rabbit model of mild, reproducible disc degeneration by an anulus needle puncture: correlation between the degree of disc injury and radiological and histological appearances of disc degeneration. Spine.

[ref-26] Messner M, Ghadge SK, Maurer T, Graber M, Staggl S, Christine Maier S, Pölzl G, Zaruba MM (2020). ZMPSTE24 is associated with elevated inflammation and progerin mRNA. Cells.

[ref-27] Nixon J (1986). Intervertebral disc mechanics: a review. Journal of the Royal Society of Medicine.

[ref-28] Odinammadu KO, Shilagardi K, Tuminelli K, Judge DP, Gordon LB, Michaelis S (2023). The farnesyl transferase inhibitor (FTI) lonafarnib improves nuclear morphology in ZMPSTE24-deficient fibroblasts from patients with the progeroid disorder MAD-B. Nucleus.

[ref-29] Poletto DL, Crowley JD, Tanglay O, Walsh WR, Pelletier MH (2023). Preclinical *in vivo* animal models of intervertebral disc degeneration. Part 1: a systematic review. The Journal of Orthopaedic Research Spine.

[ref-30] Quigley A, Dong YY, Pike AC, Dong L, Shrestha L, Berridge G, Stansfeld PJ, Sansom MS, Edwards AM, Bountra C, Von Delft F, Bullock AN, Burgess-Brown NA, Carpenter EP (2013). The structural basis of ZMPSTE24-dependent laminopathies. Science.

[ref-31] Ragnauth CD, Warren DT, Liu Y, McNair R, Tajsic T, Figg N, Shroff R, Skepper J, Shanahan CM (2010). Prelamin A acts to accelerate smooth muscle cell senescence and is a novel biomarker of human vascular aging. Circulation.

[ref-32] Sieprath T, Corne TD, Nooteboom M, Grootaert C, Rajkovic A, Buysschaert B, Robijns J, Broers JL, Ramaekers FC, Koopman WJ, Willems PH, De Vos WH (2015). Sustained accumulation of prelamin A and depletion of lamin A/C both cause oxidative stress and mitochondrial dysfunction but induce different cell fates. Nucleus.

[ref-33] Singh K, Masuda K, An HS (2005). Animal models for human disc degeneration. The Spine Journal.

[ref-34] Song C, Zhou Y, Cheng K, Liu F, Cai W, Zhou D, Chen R, Shi H, Fu Z, Chen J, Liu Z (2023). Cellular senescence - molecular mechanisms of intervertebral disc degeneration from an immune perspective. Biomedicine & Pharmacotherapy.

[ref-35] Sun K, Jiang J, Wang Y, Sun X, Zhu J, Xu X, Sun J, Shi J (2022). The role of nerve fibers and their neurotransmitters in regulating intervertebral disc degeneration. Ageing Research Reviews.

[ref-36] Suo J, Shao R, Yang R, Wang J, Zhang Z, Wang D, Niu N, Zheng X, Zou W (2023). Accelerated aging in articular cartilage by ZMPSTE24 deficiency leads to osteoarthritis with impaired metabolic signaling and epigenetic regulation. Cell Death & Disease.

[ref-37] Tong W, Lu Z, Qin L, Mauck RL, Smith HE, Smith LJ, Malhotra NR, Heyworth MF, Caldera F, Enomoto-Iwamoto M, Zhang Y (2017). Cell therapy for the degenerating intervertebral disc. Translational Research.

[ref-38] Tripathi G, Bhombe K, Kumar H (2025). Backbone breakthroughs: how rodent models are shaping intervertebral disc disease treatment. The Journal of Pain.

[ref-39] Worman HJ, Michaelis S (2023). Prelamin A and ZMPSTE24 in premature and physiological aging. Nucleus.

[ref-40] Wu D, Flannery AR, Cai H, Ko E, Cao K (2014). Nuclear localization signal deletion mutants of lamin A and progerin reveal insights into lamin A processing and emerin targeting. Nucleus.

[ref-41] Yang R, Cao D, Suo J, Zhang L, Mo C, Wang M, Niu N, Yue R, Zou W (2023). Premature aging of skeletal stem/progenitor cells rather than osteoblasts causes bone loss with decreased mechanosensation. Bone Research.

[ref-42] Yang S, Zhang Y, Peng Q, Meng B, Wang J, Sun H, Chen L, Dai R, Zhang L (2024). Regulating pyroptosis by mesenchymal stem cells and extracellular vesicles: a promising strategy to alleviate intervertebral disc degeneration. Biomedicine & Pharmacotherapy.

[ref-43] Zhao CQ, Wang LM, Jiang LS, Dai LY (2007). The cell biology of intervertebral disc aging and degeneration. Ageing Research Reviews.

[ref-44] Zhu Y, Tchkonia T, Pirtskhalava T, Gower AC, Ding H, Giorgadze N, Palmer AK, Ikeno Y, Hubbard GB, Lenburg M, O’Hara SP, LaRusso NF, Miller JD, Roos CM, Verzosa GC, LeBrasseur NK, Wren JD, Farr JN, Khosla S, Stout MB, McGowan SJ, Fuhrmann-Stroissnigg H, Gurkar AU, Zhao J, Colangelo D, Dorronsoro A, Ling YY, Barghouthy AS, Navarro DC, Sano T, Robbins PD, Niedernhofer LJ, Kirkland JL (2015). The Achilles’ heel of senescent cells: from transcriptome to senolytic drugs. Aging Cell.

