# Supplementary figures and images for: Zmpste24 deficiency contributes to intervertebral disc degeneration by undermining the stability of the nuclear membrane of nucleus pulposus cells
Source: PeerJ. 2026 Jan 7;14:e20534. doi: 10.7717/peerj.20534 (PMC12790285; doi:10.7717/peerj.20534)

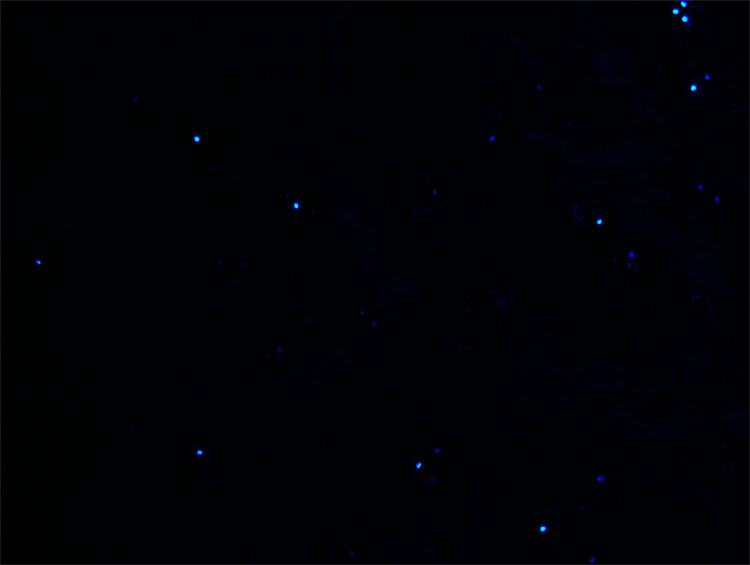

Supplement: Supplemental Information 1 [file peerj-14-20534-s001.zip › raw data1/figure1/3 4 dapi.jpg]

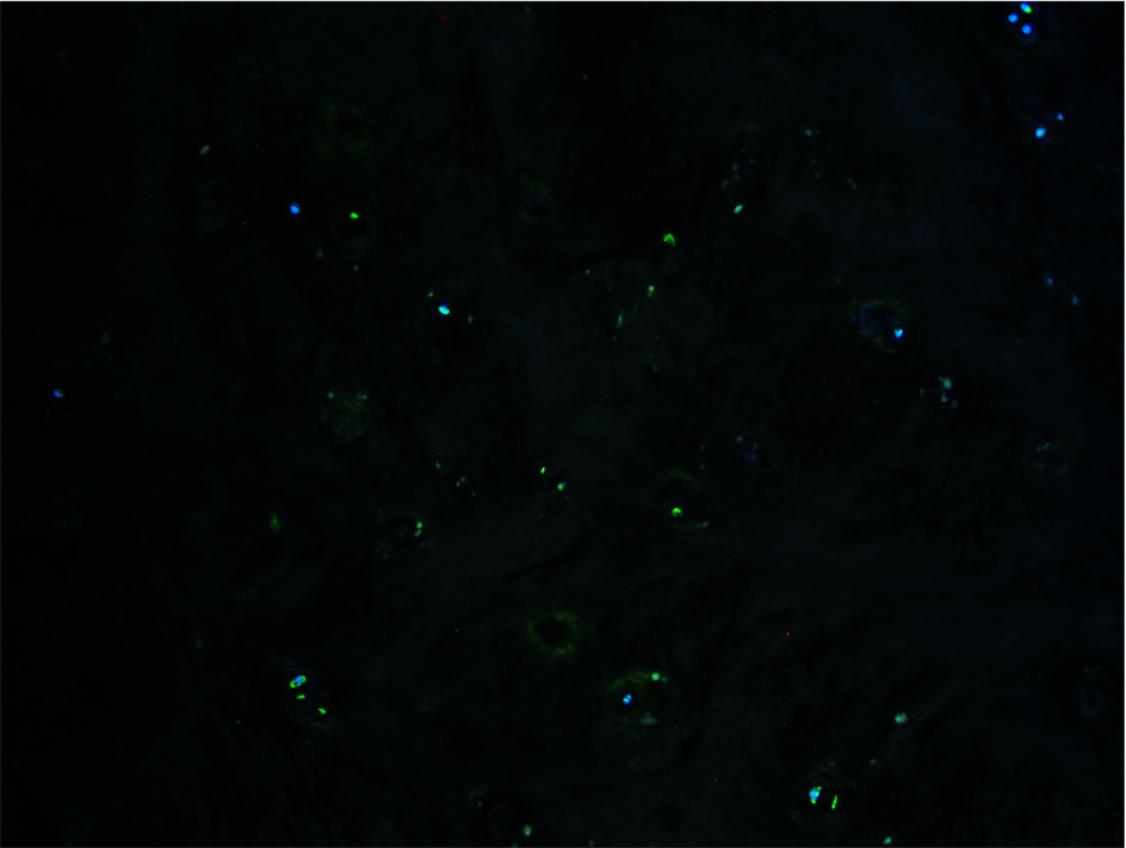

Supplement: Supplemental Information 1 [file peerj-14-20534-s001.zip › raw data1/figure1/3 4 merge.jpg]

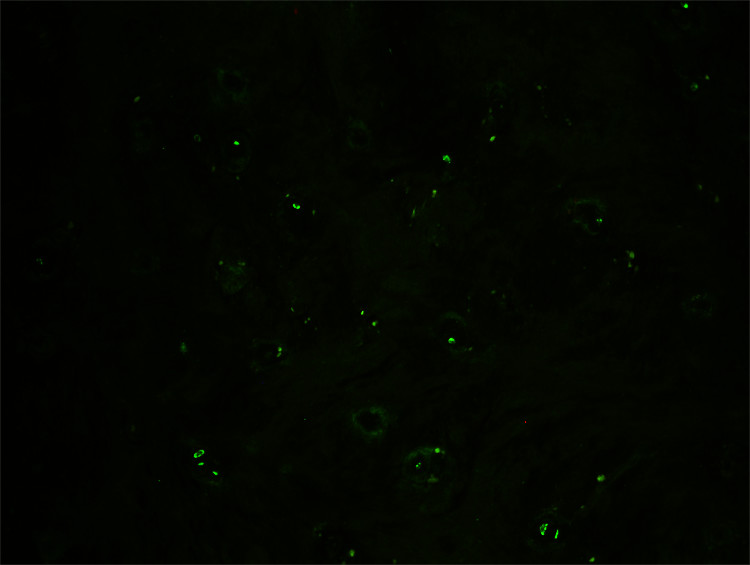

Supplement: Supplemental Information 1 [file peerj-14-20534-s001.zip › raw data1/figure1/3 4 zmp.jpg]

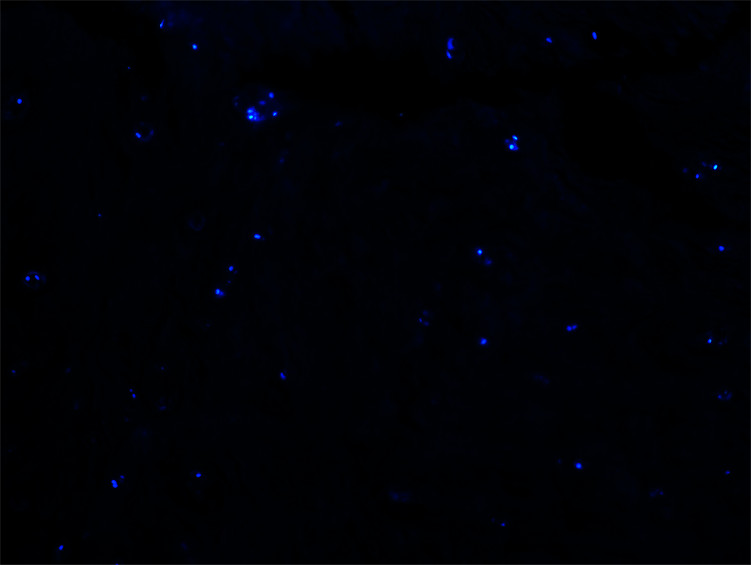

Supplement: Supplemental Information 1 [file peerj-14-20534-s001.zip › raw data1/figure1/5 6 dapi.jpg]

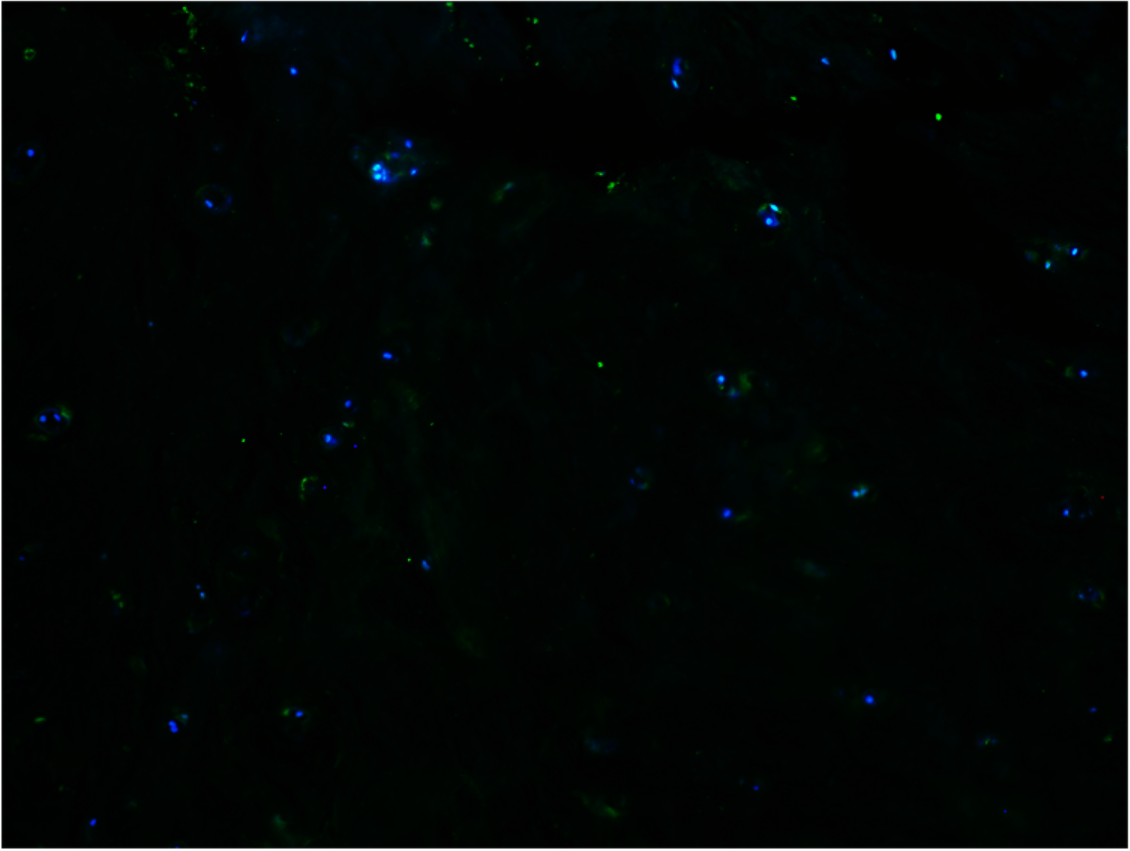

Supplement: Supplemental Information 1 [file peerj-14-20534-s001.zip › raw data1/figure1/5 6 merge.jpg]

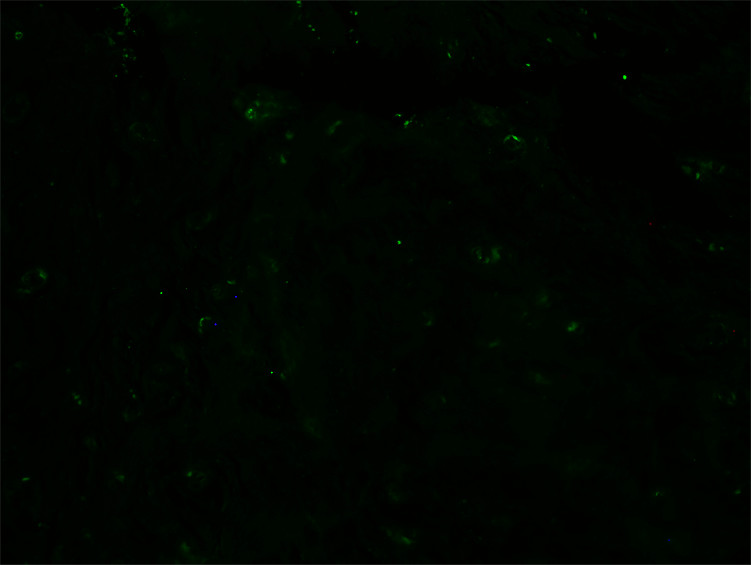

Supplement: Supplemental Information 1 [file peerj-14-20534-s001.zip › raw data1/figure1/5 6 zmp.jpg]

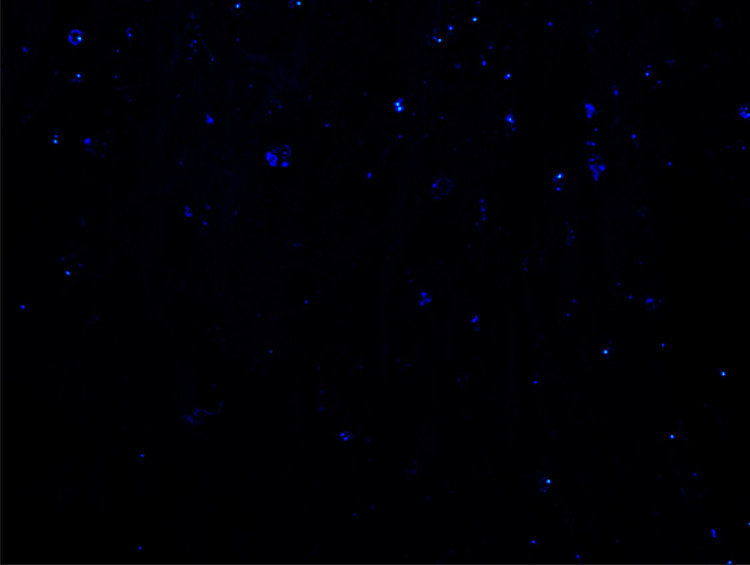

Supplement: Supplemental Information 1 [file peerj-14-20534-s001.zip › raw data1/figure1/7 8 dapi .jpg]

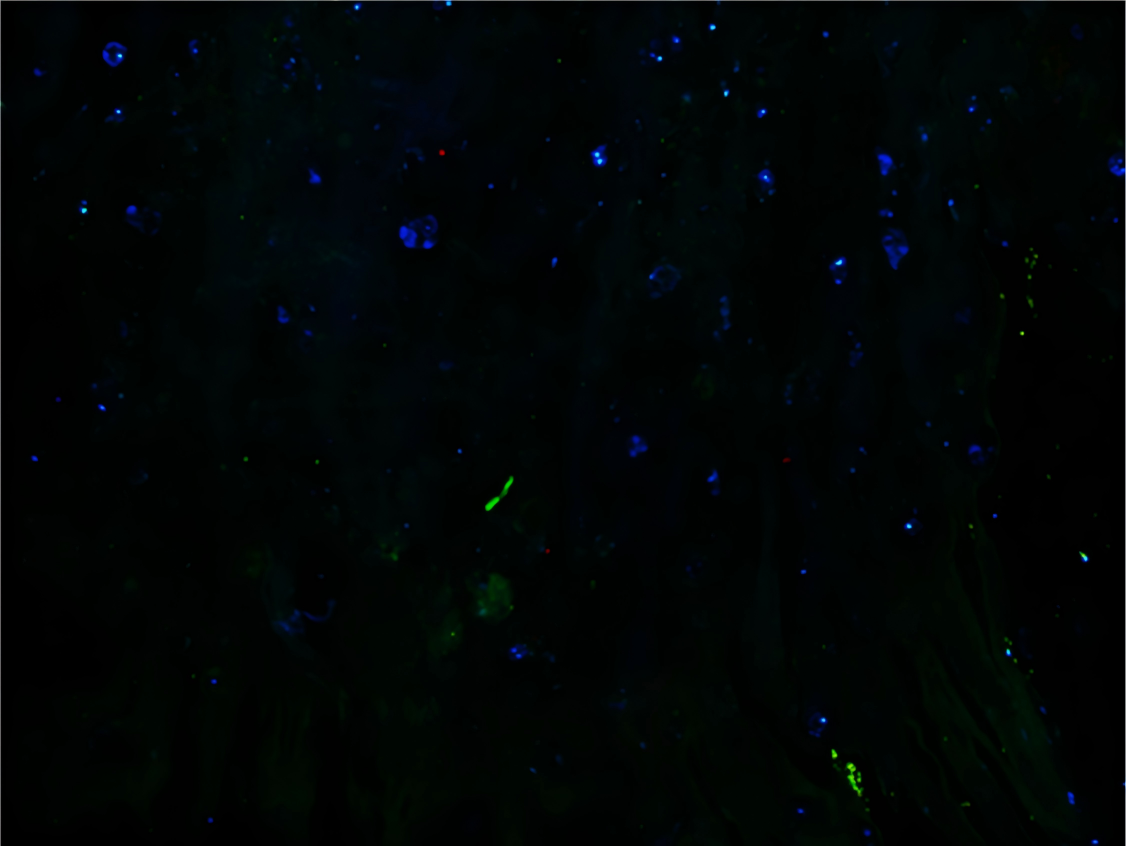

Supplement: Supplemental Information 1 [file peerj-14-20534-s001.zip › raw data1/figure1/7 8 merge.jpg]

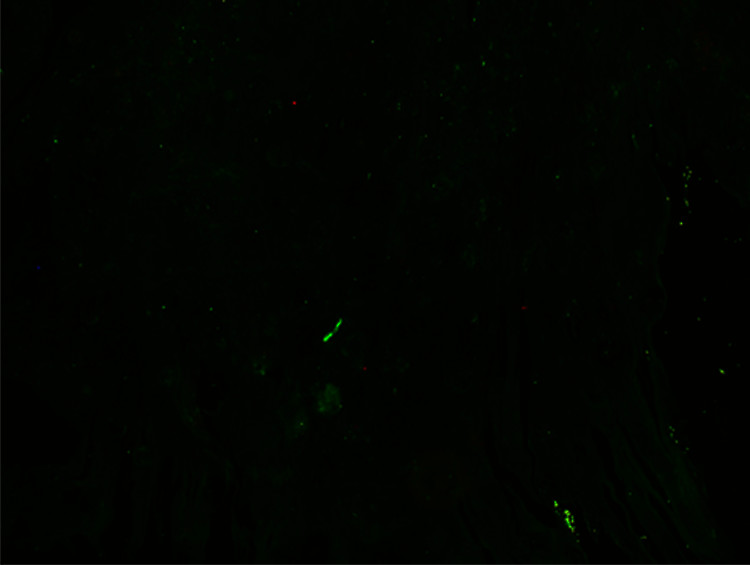

Supplement: Supplemental Information 1 [file peerj-14-20534-s001.zip › raw data1/figure1/7 8 zmp .jpg]

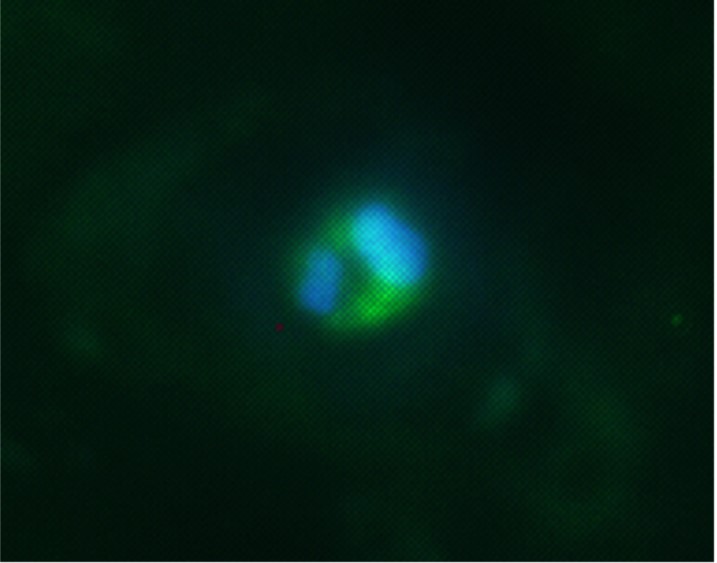

Supplement: Supplemental Information 1 [file peerj-14-20534-s001.zip › raw data1/figure1/Figure 1 C 1.jpg]

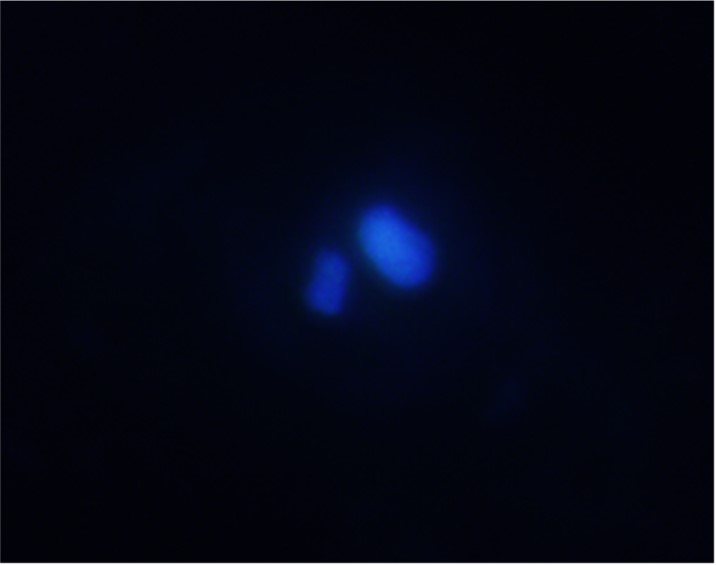

Supplement: Supplemental Information 1 [file peerj-14-20534-s001.zip › raw data1/figure1/Figure 1 C 2.jpg]

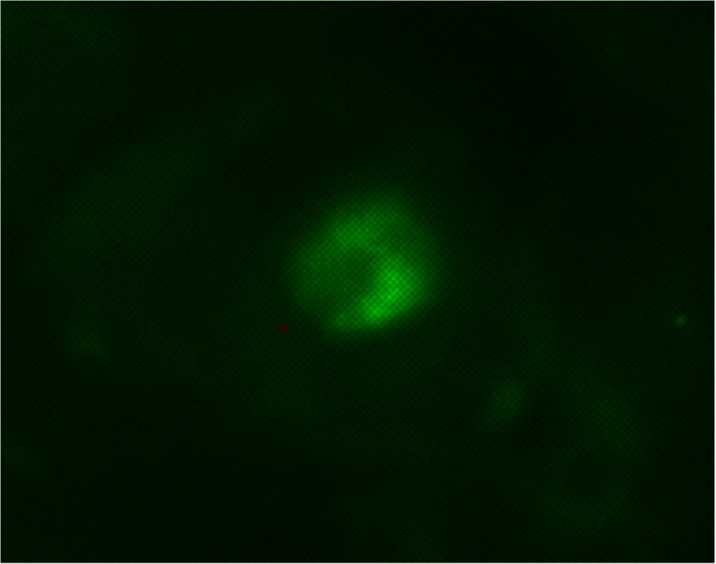

Supplement: Supplemental Information 1 [file peerj-14-20534-s001.zip › raw data1/figure1/Figure 1 C 3.jpg]

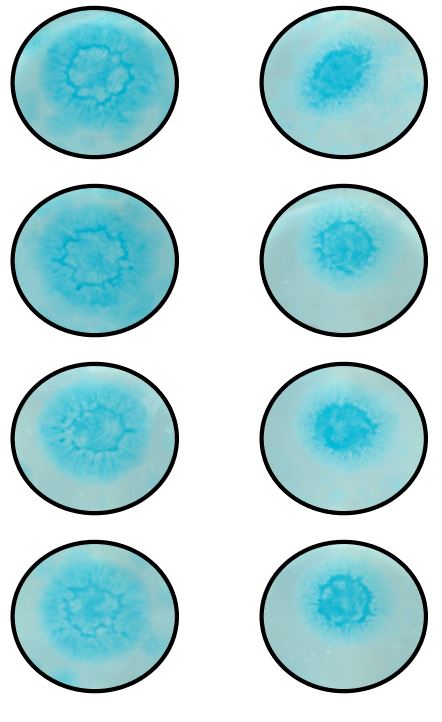

Supplement: Supplemental Information 1 [file peerj-14-20534-s001.zip › raw data1/figure2/Figure 2 A.jpg]

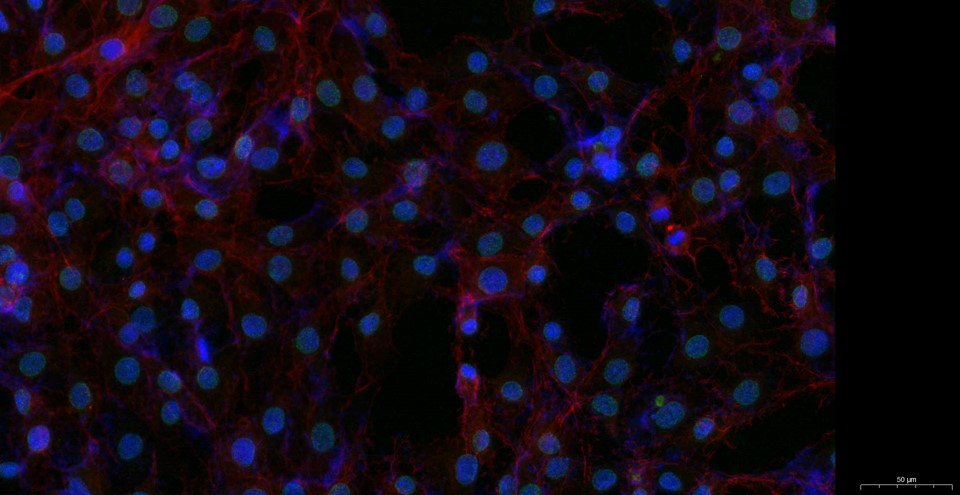

Supplement: Supplemental Information 1 [file peerj-14-20534-s001.zip › raw data1/figure2/Figure 2 G Lopinavir 1.jpg]

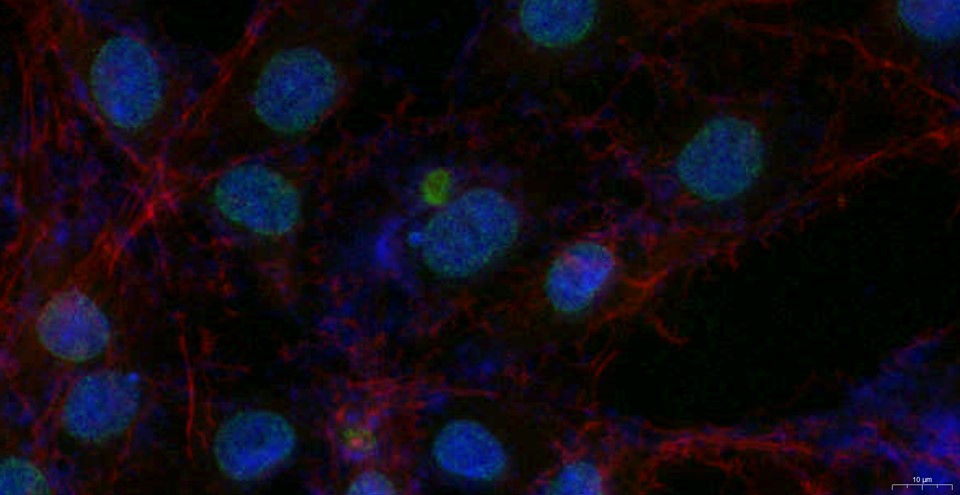

Supplement: Supplemental Information 1 [file peerj-14-20534-s001.zip › raw data1/figure2/Figure 2 G Lopinavir 2.jpg]

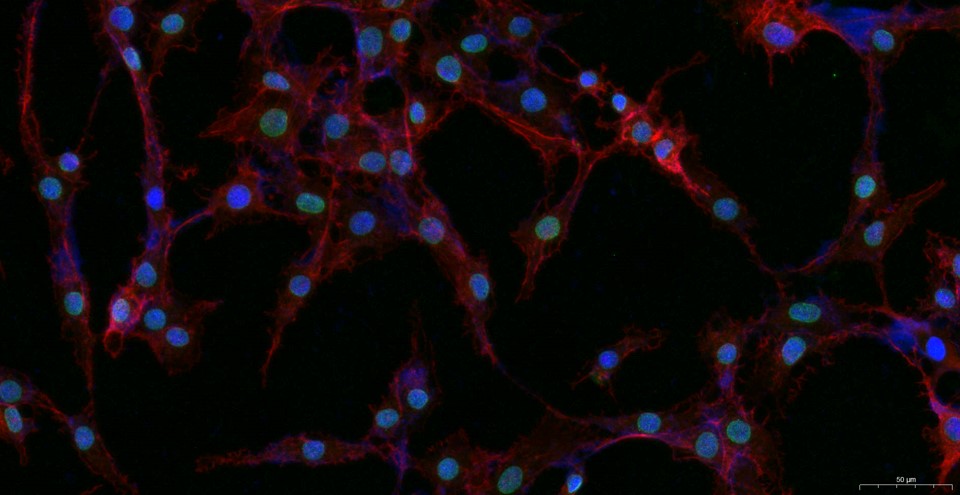

Supplement: Supplemental Information 1 [file peerj-14-20534-s001.zip › raw data1/figure2/Figure 2 G control 1.jpg]

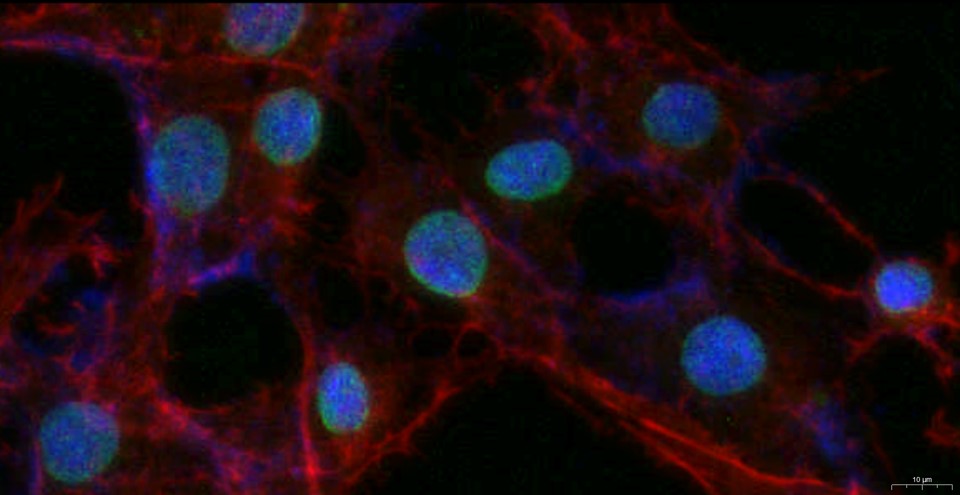

Supplement: Supplemental Information 1 [file peerj-14-20534-s001.zip › raw data1/figure2/Figure 2 G control 2.jpg]

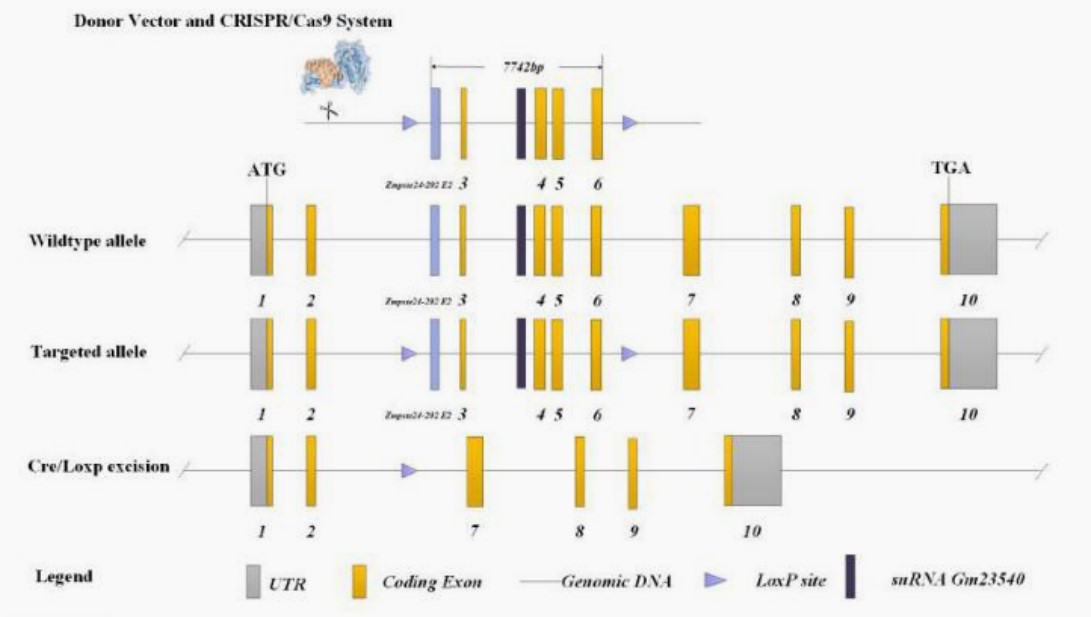

Supplement: Supplemental Information 2 [file peerj-14-20534-s002.zip › raw data2/figure3/Figure 3 A.jpg]

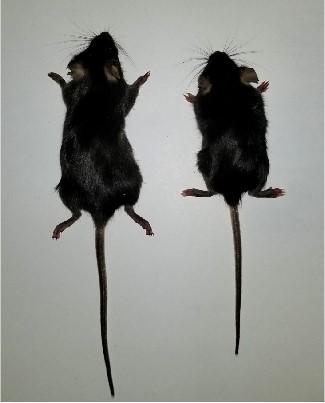

Supplement: Supplemental Information 2 [file peerj-14-20534-s002.zip › raw data2/figure3/Figure 3 B.jpg]

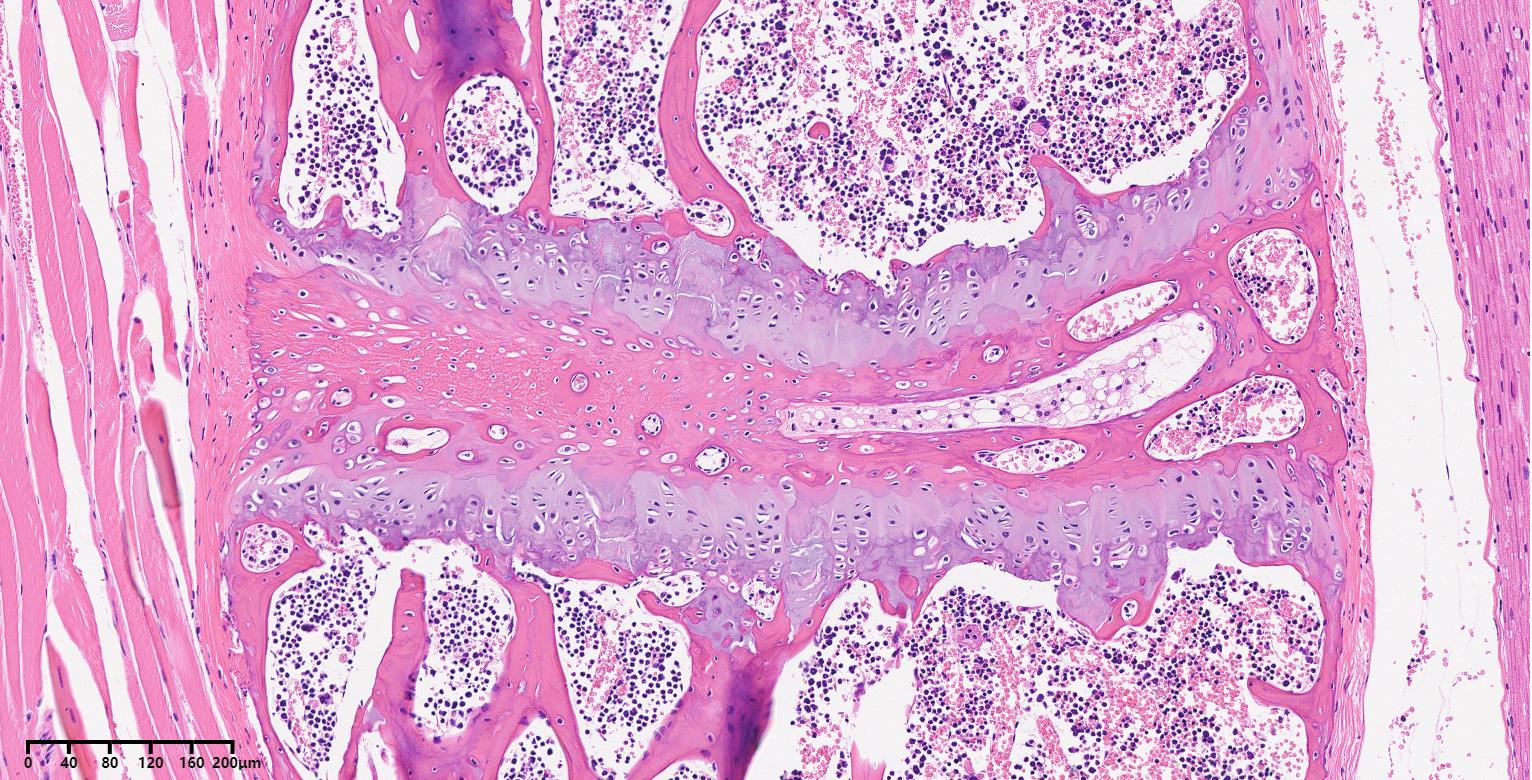

Supplement: Supplemental Information 3 [file peerj-14-20534-s003.zip › raw data3/figure4/Figure 4 A ko 2.jpg]

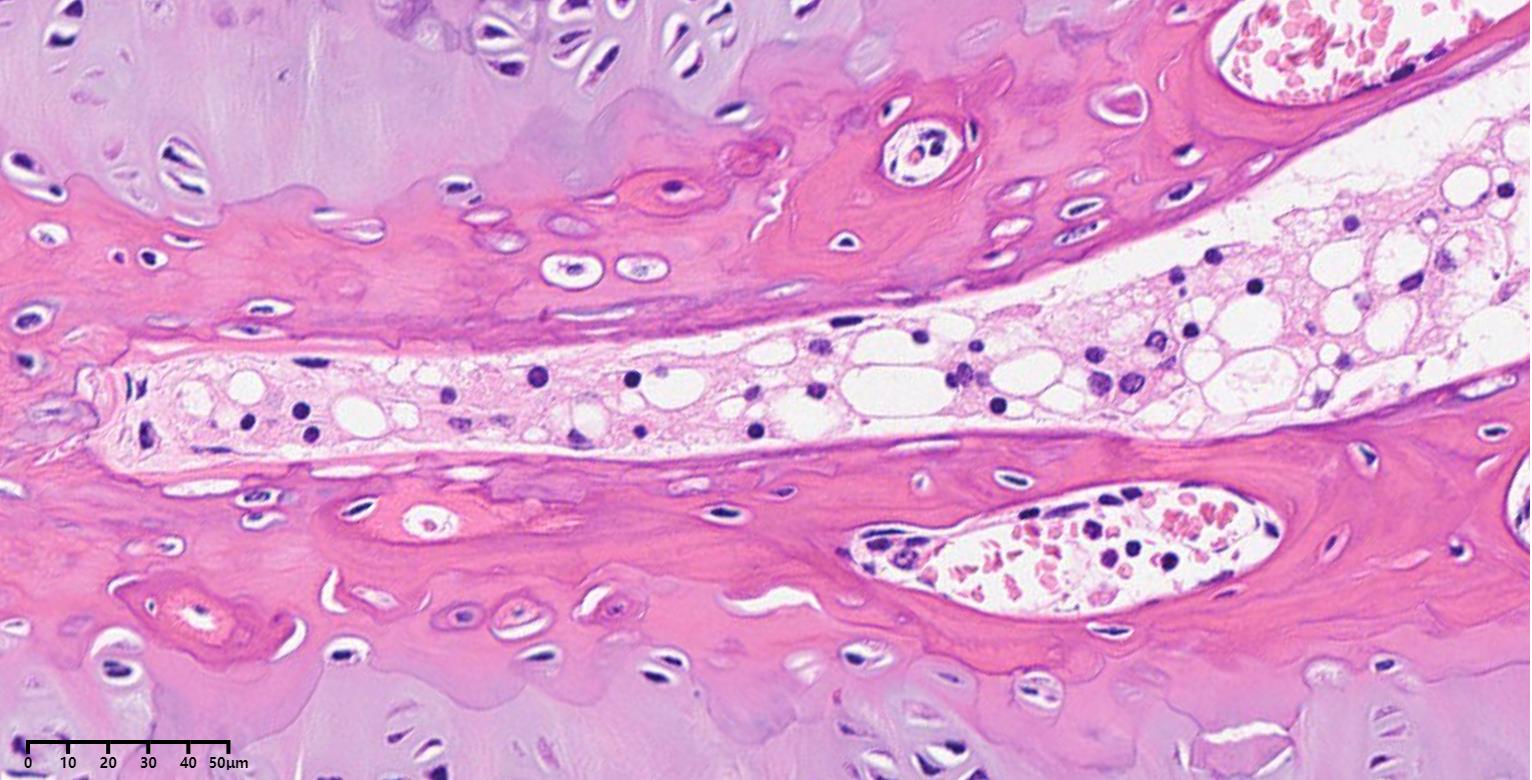

Supplement: Supplemental Information 3 [file peerj-14-20534-s003.zip › raw data3/figure4/Figure 4 A ko1 .jpg]

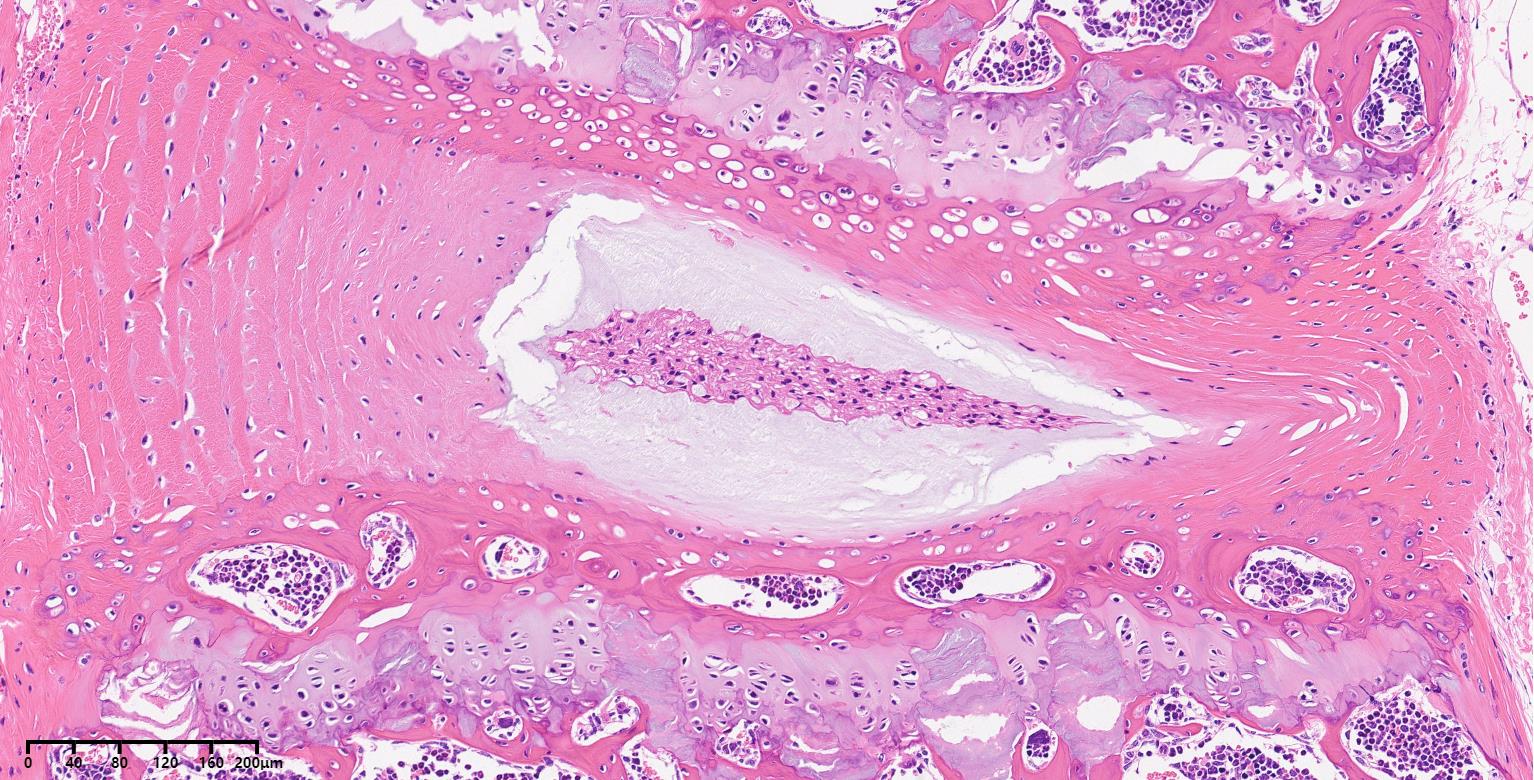

Supplement: Supplemental Information 3 [file peerj-14-20534-s003.zip › raw data3/figure4/Figure 4 A kw 1.jpg]

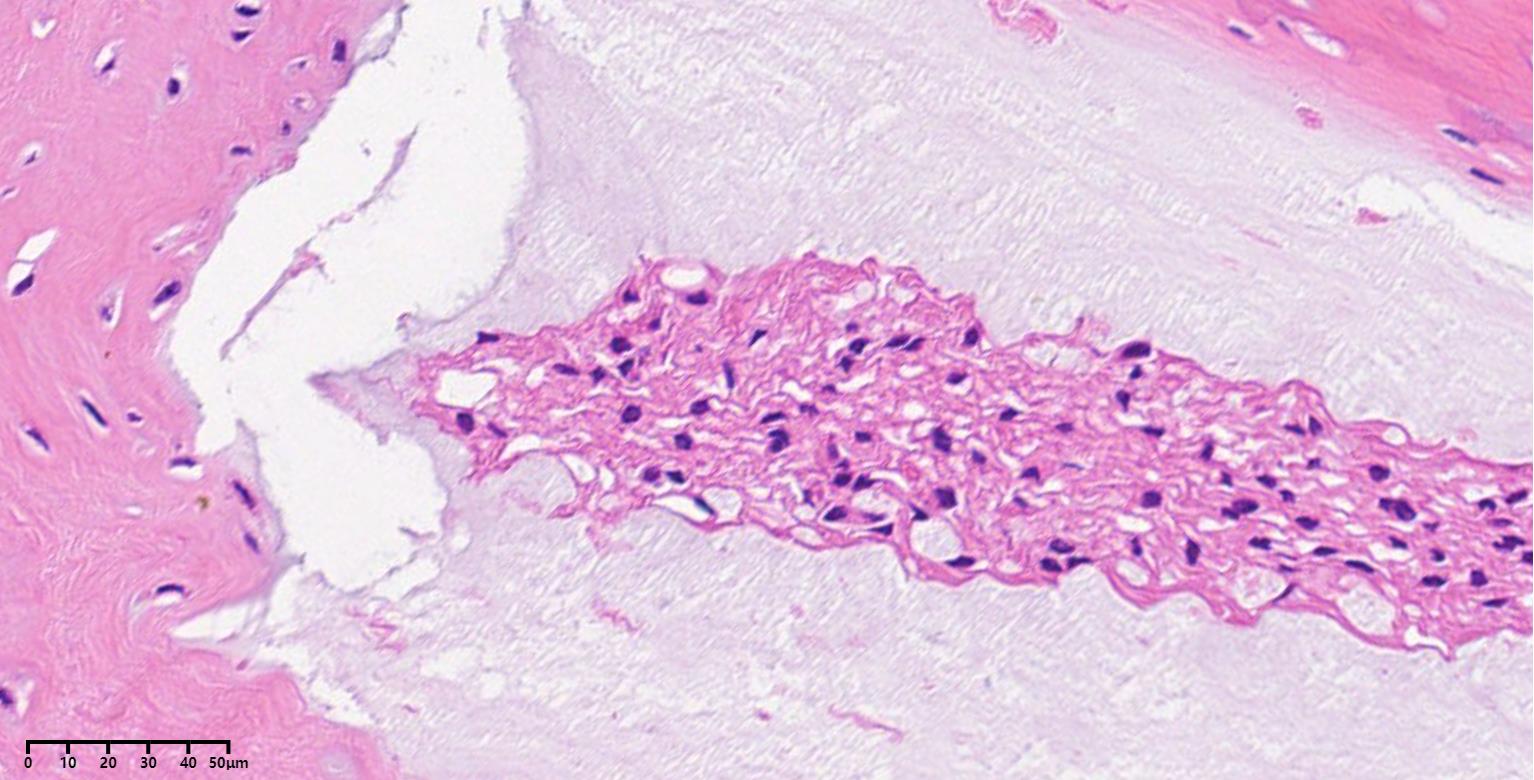

Supplement: Supplemental Information 3 [file peerj-14-20534-s003.zip › raw data3/figure4/Figure 4 A kw 2.jpg]

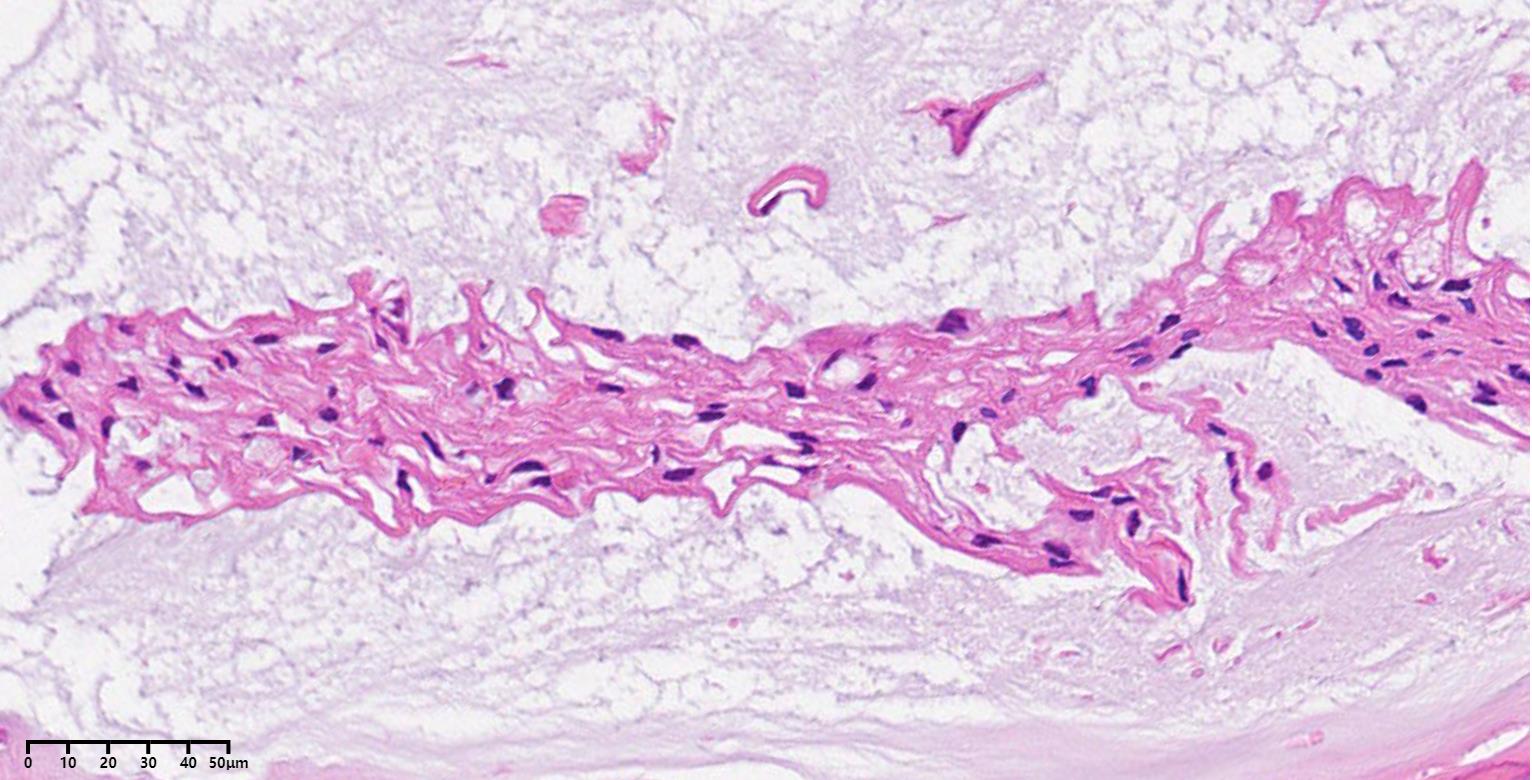

Supplement: Supplemental Information 3 [file peerj-14-20534-s003.zip › raw data3/figure4/Figure 4 A wt 2.jpg]

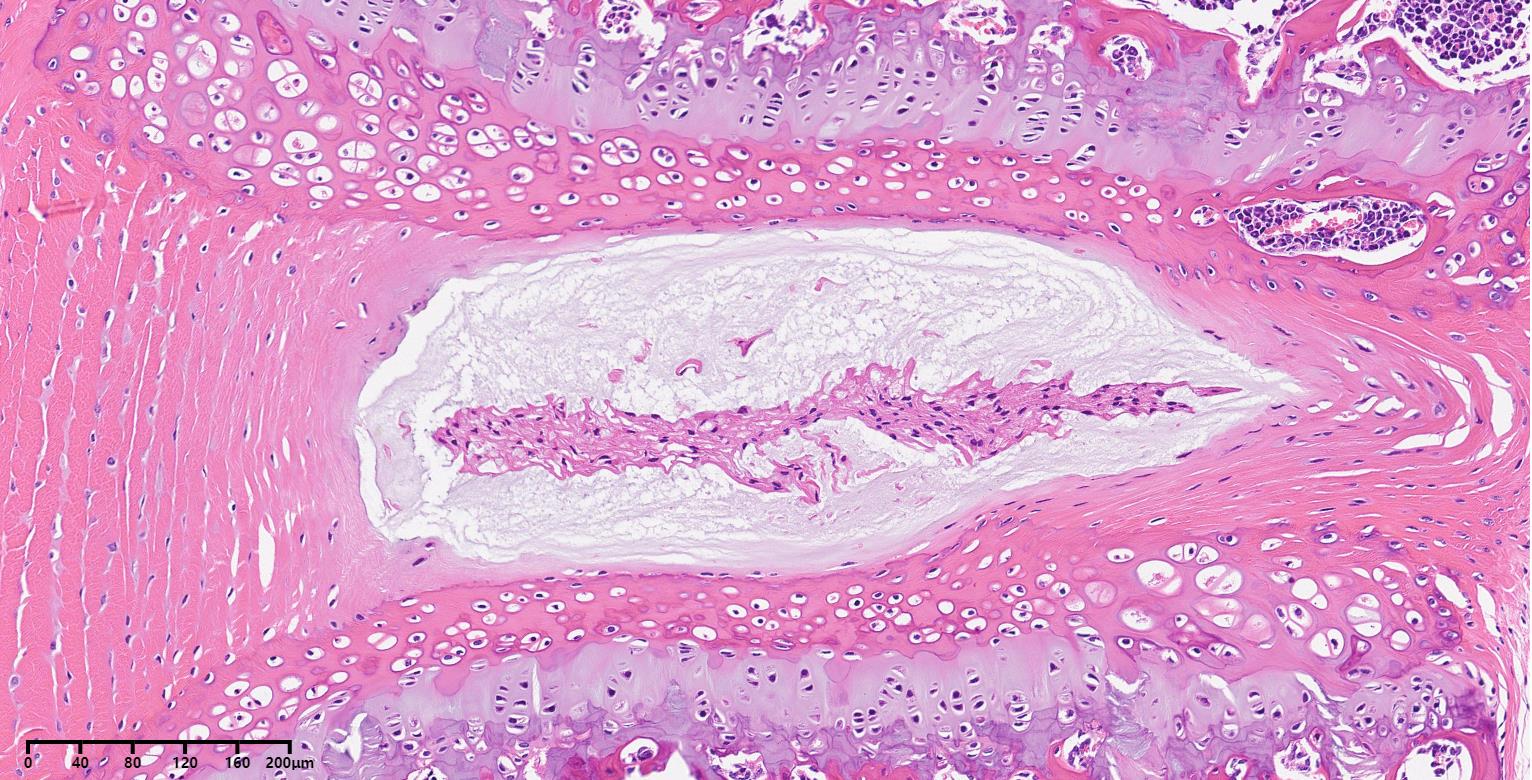

Supplement: Supplemental Information 3 [file peerj-14-20534-s003.zip › raw data3/figure4/Figure 4 A wt1.jpg]

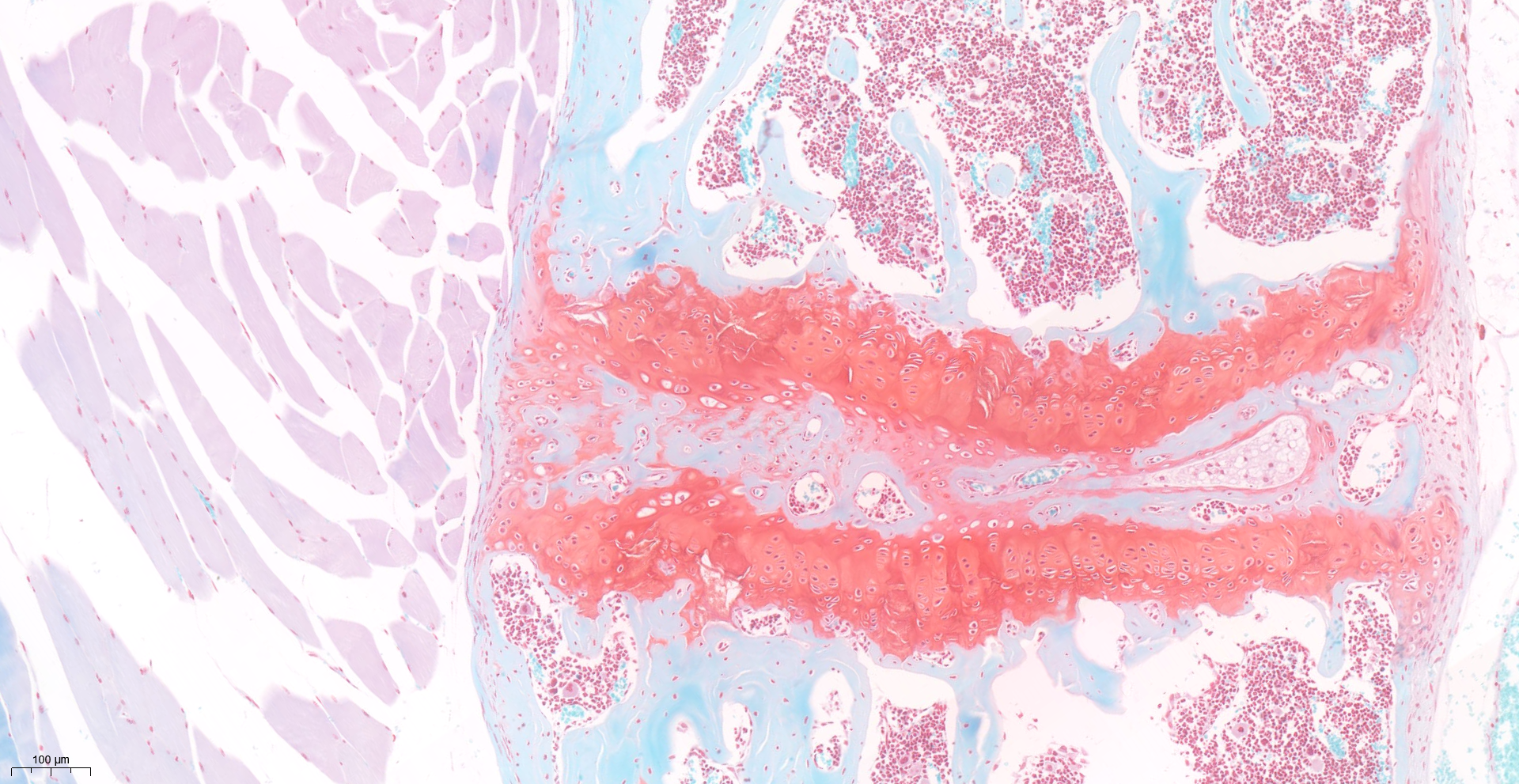

Supplement: Supplemental Information 3 [file peerj-14-20534-s003.zip › raw data3/figure4/Figure 4 C ko 1.jpg]

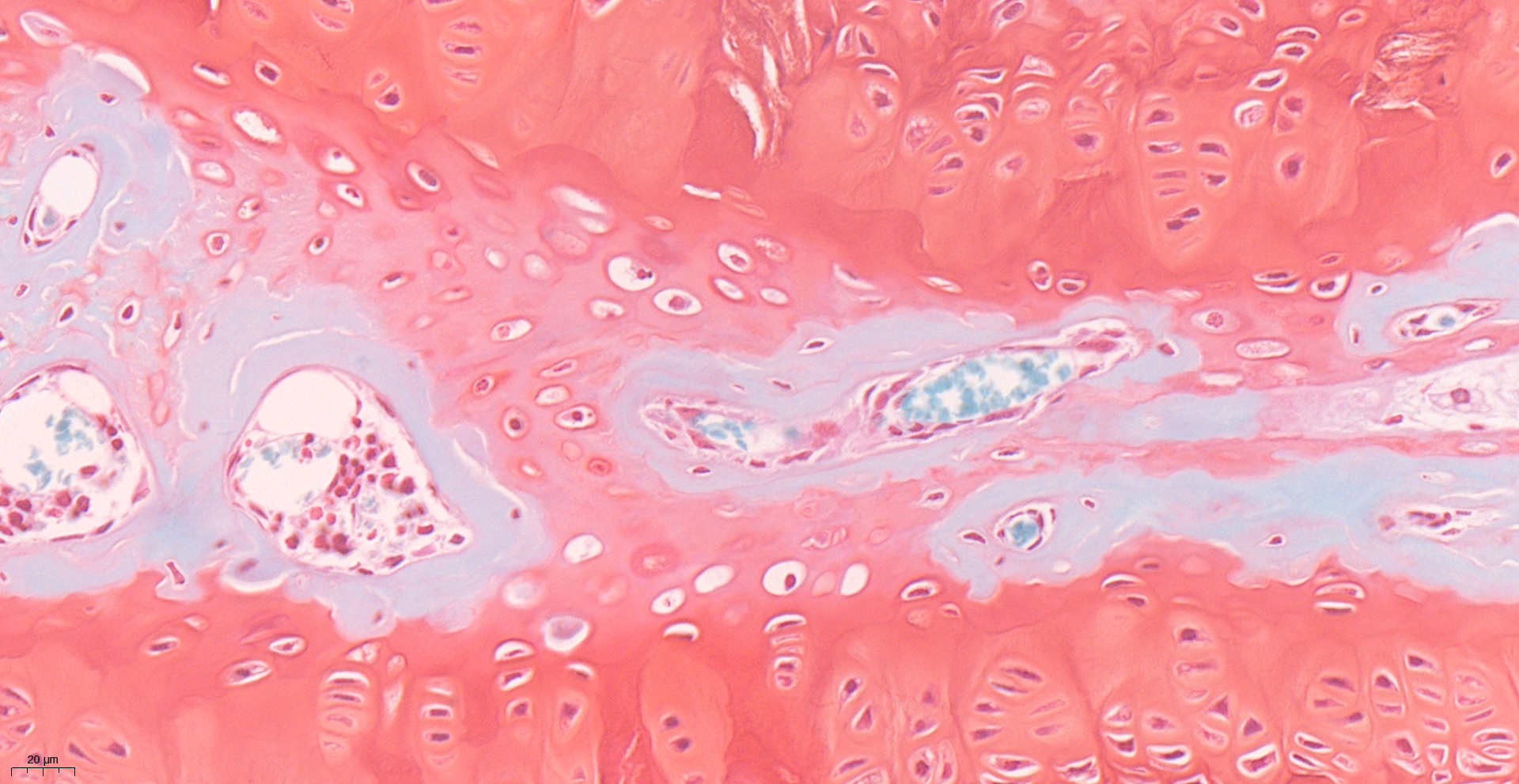

Supplement: Supplemental Information 3 [file peerj-14-20534-s003.zip › raw data3/figure4/Figure 4 C ko2.jpg]

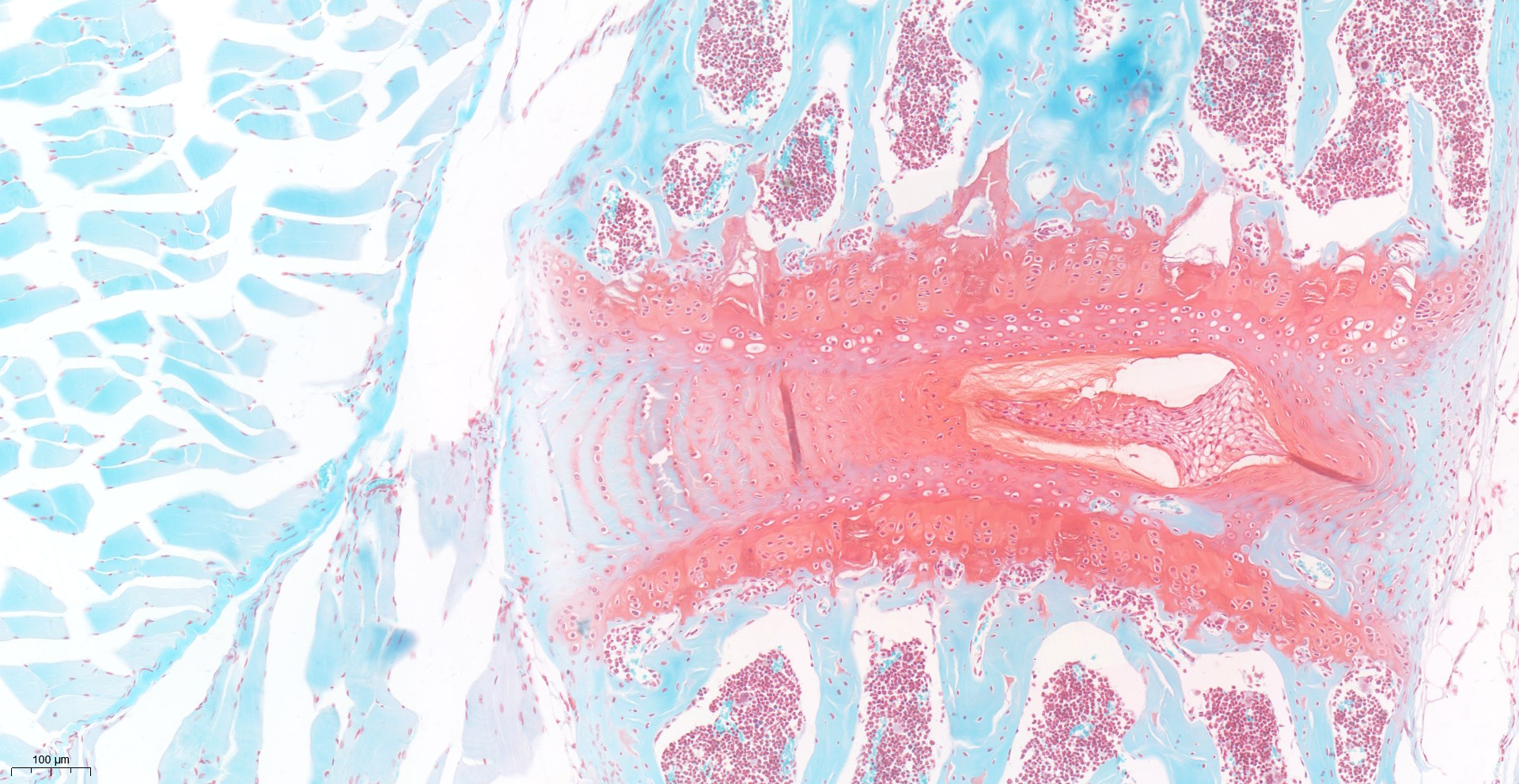

Supplement: Supplemental Information 3 [file peerj-14-20534-s003.zip › raw data3/figure4/Figure 4 C kw 1.jpg]

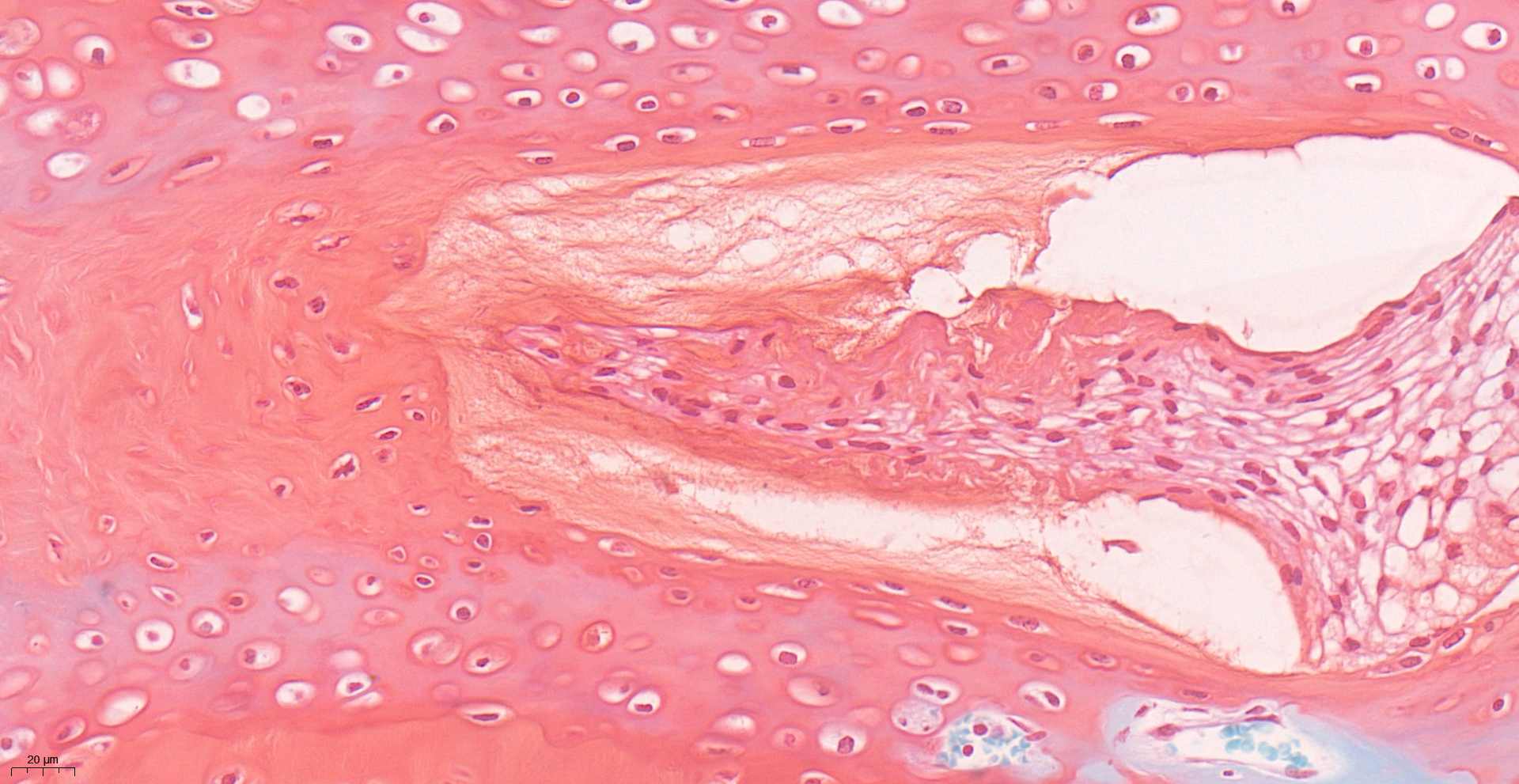

Supplement: Supplemental Information 3 [file peerj-14-20534-s003.zip › raw data3/figure4/Figure 4 C kw 2.jpg]

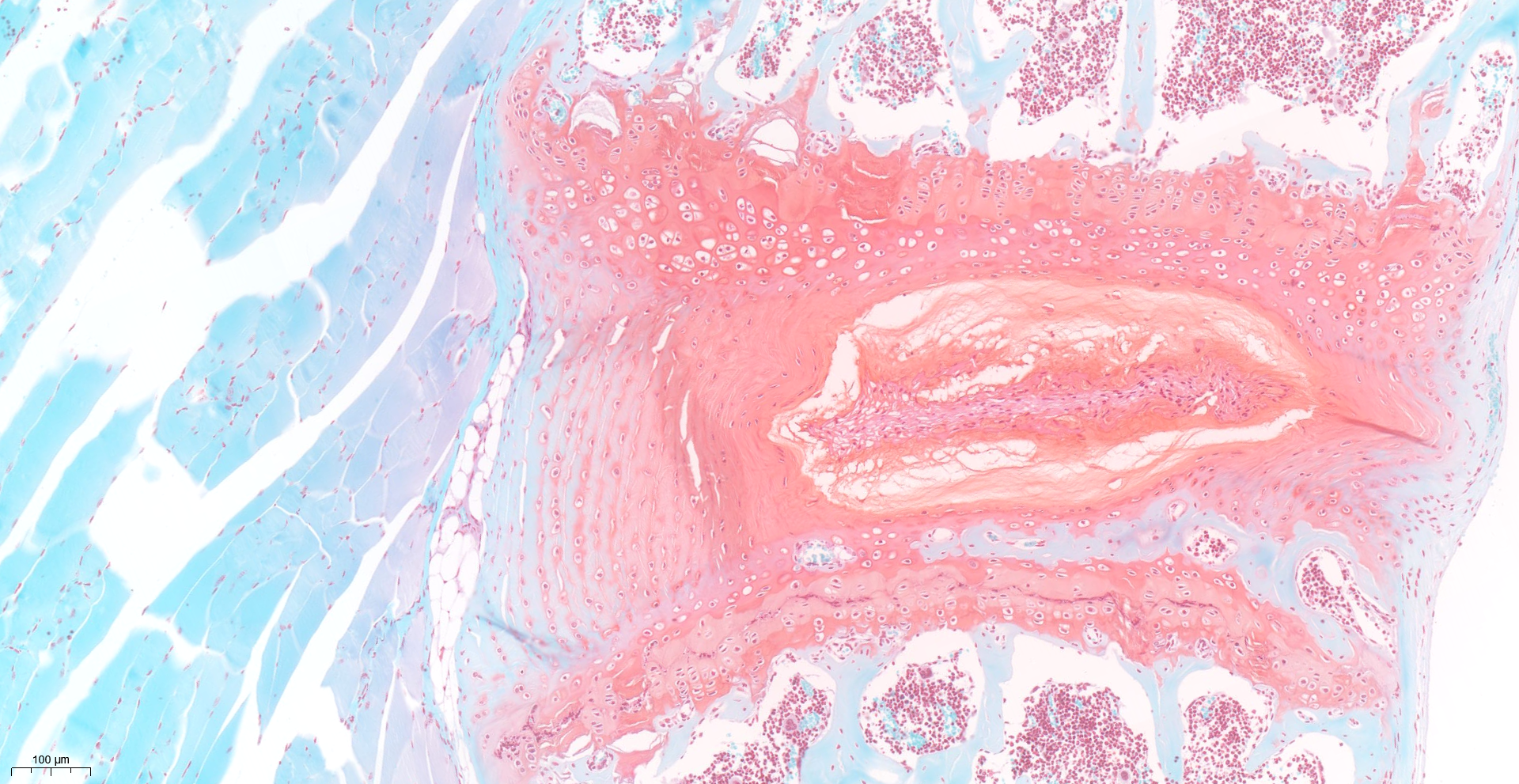

Supplement: Supplemental Information 3 [file peerj-14-20534-s003.zip › raw data3/figure4/Figure 4 C wt 1.jpg]

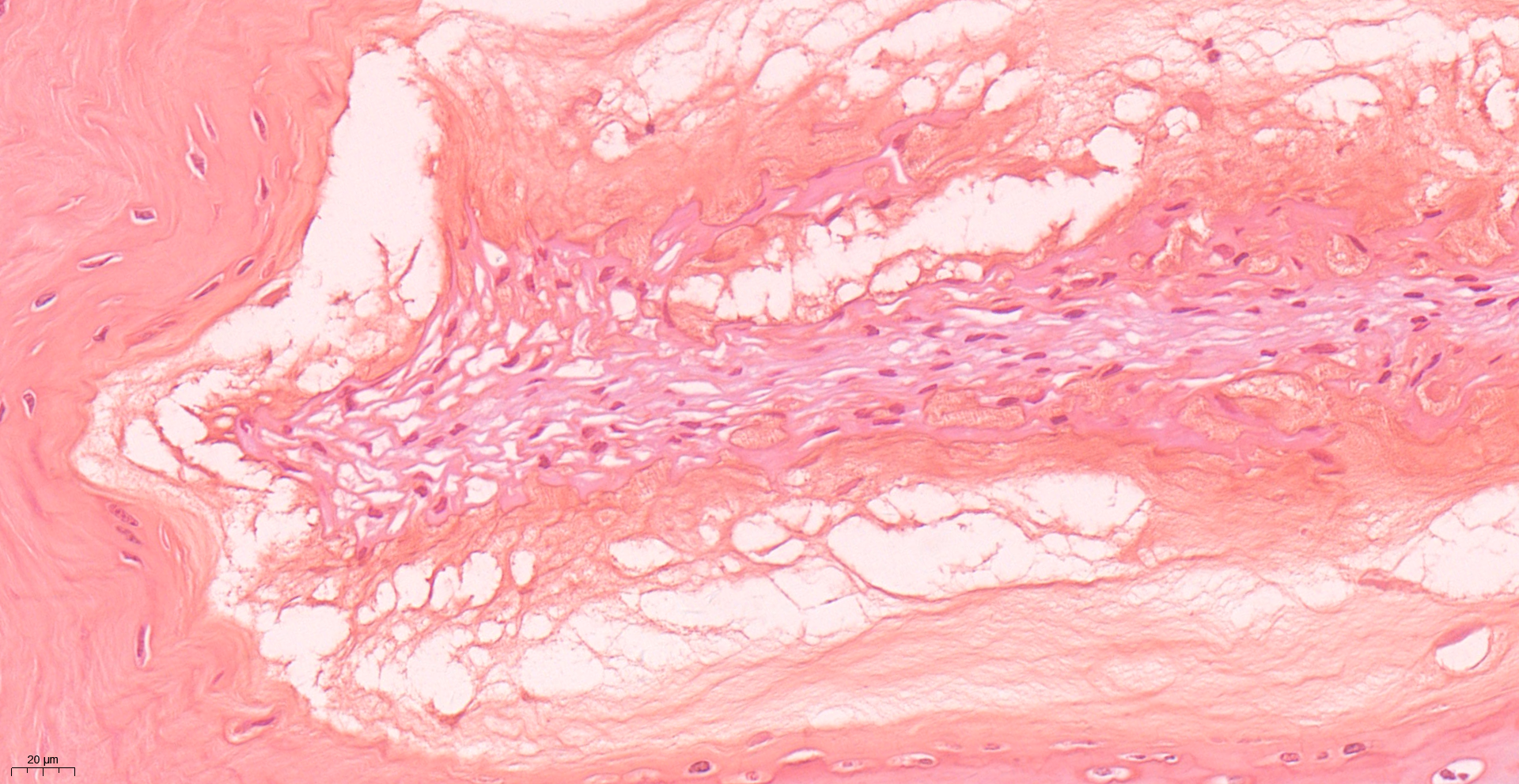

Supplement: Supplemental Information 3 [file peerj-14-20534-s003.zip › raw data3/figure4/Figure 4 C wt 2.jpg]

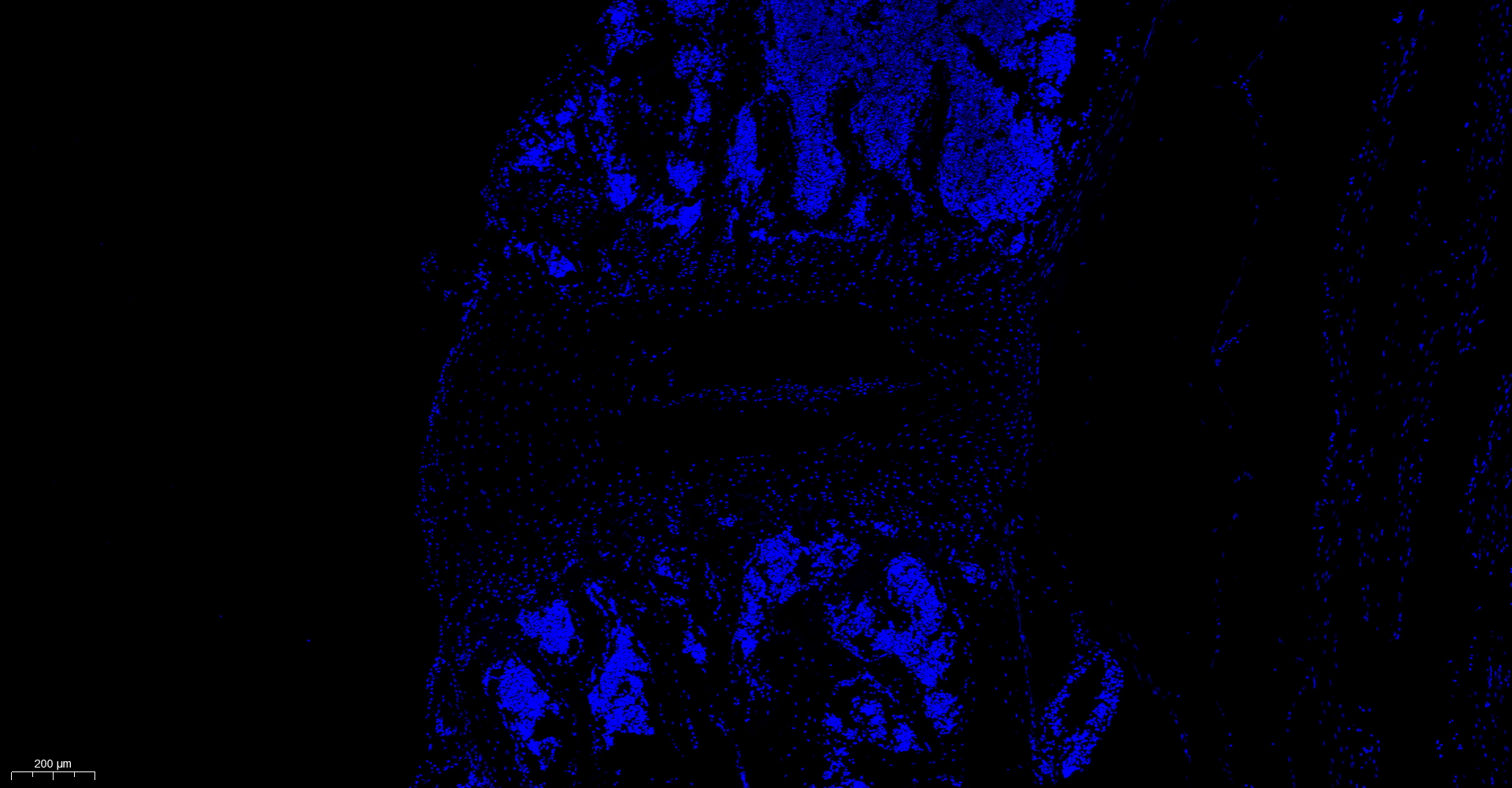

Supplement: Supplemental Information 4 [file peerj-14-20534-s004.zip › raw data4/Figure 4 D1.jpg]

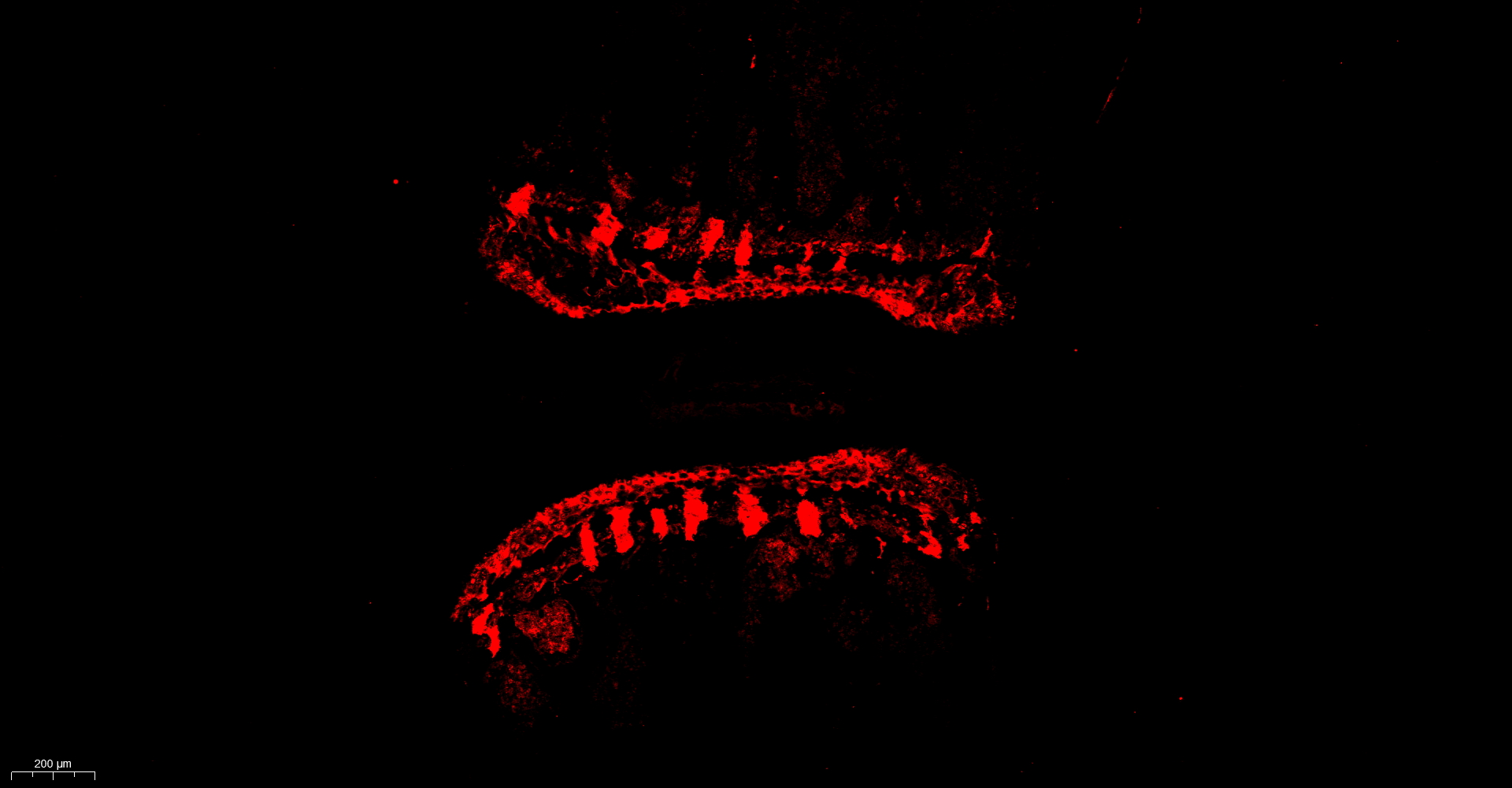

Supplement: Supplemental Information 4 [file peerj-14-20534-s004.zip › raw data4/Figure 4 D2.jpg]

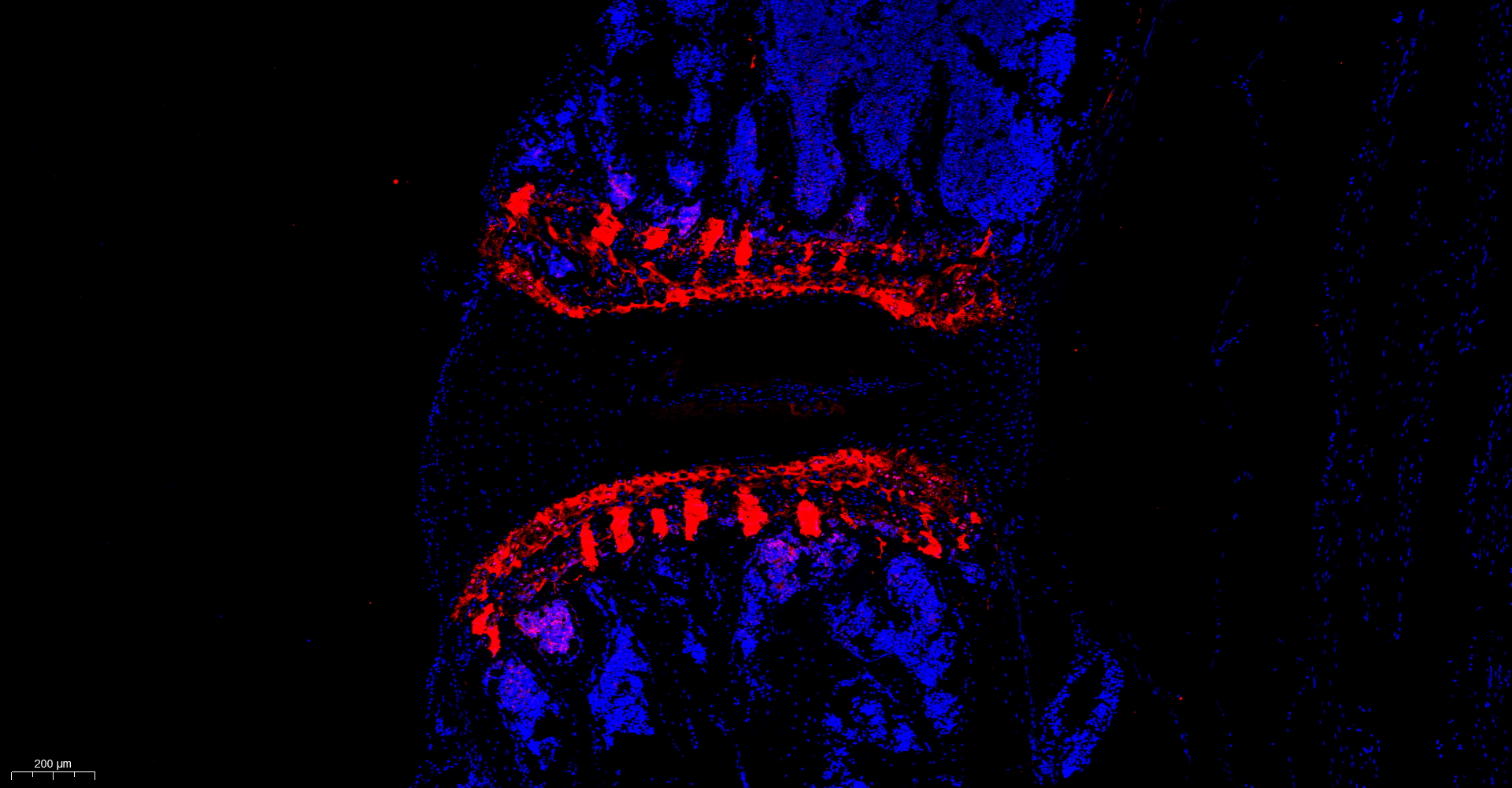

Supplement: Supplemental Information 4 [file peerj-14-20534-s004.zip › raw data4/Figure 4 D3.jpg]

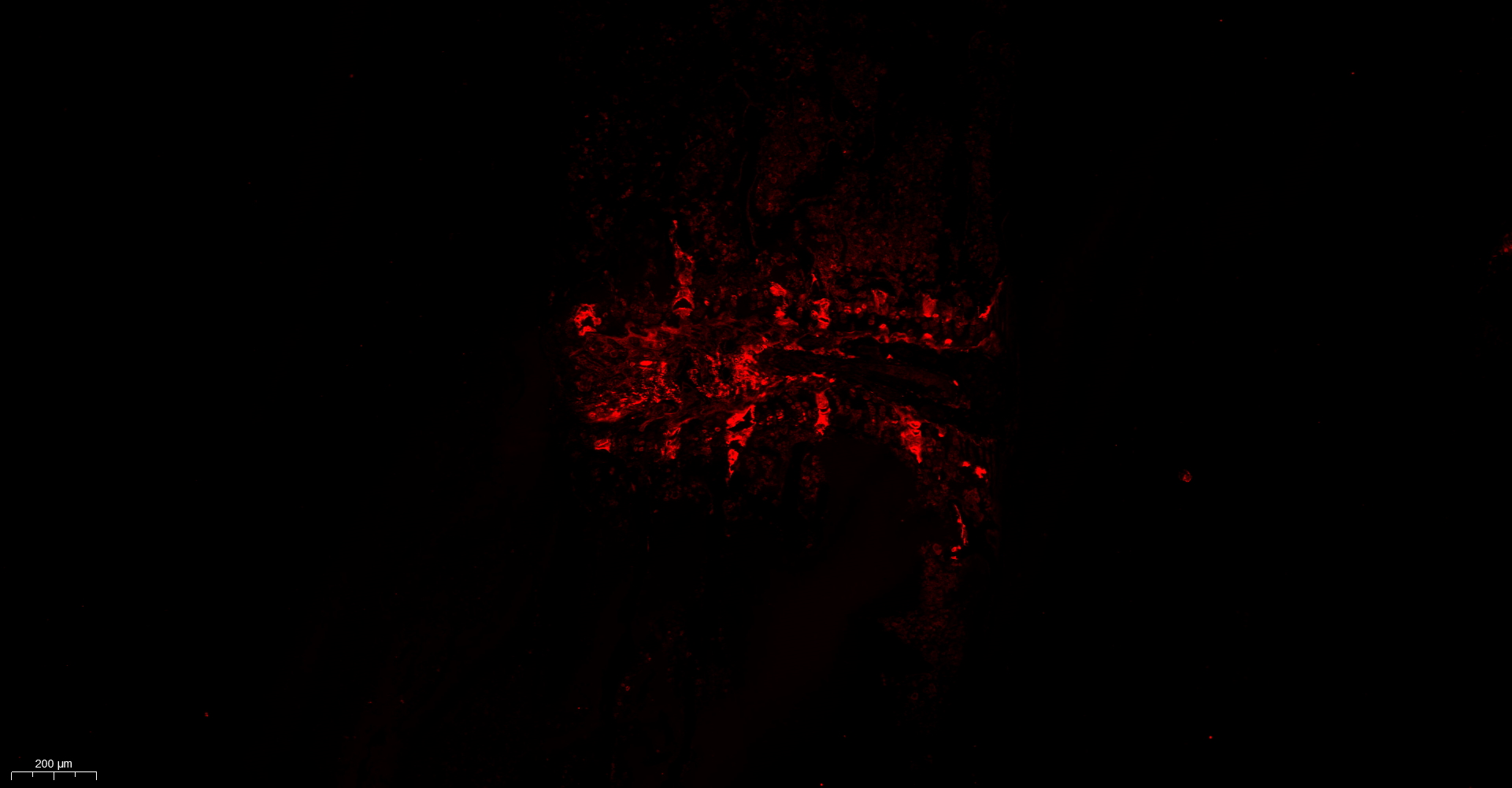

Supplement: Supplemental Information 4 [file peerj-14-20534-s004.zip › raw data4/Figure 4 D4.jpg]

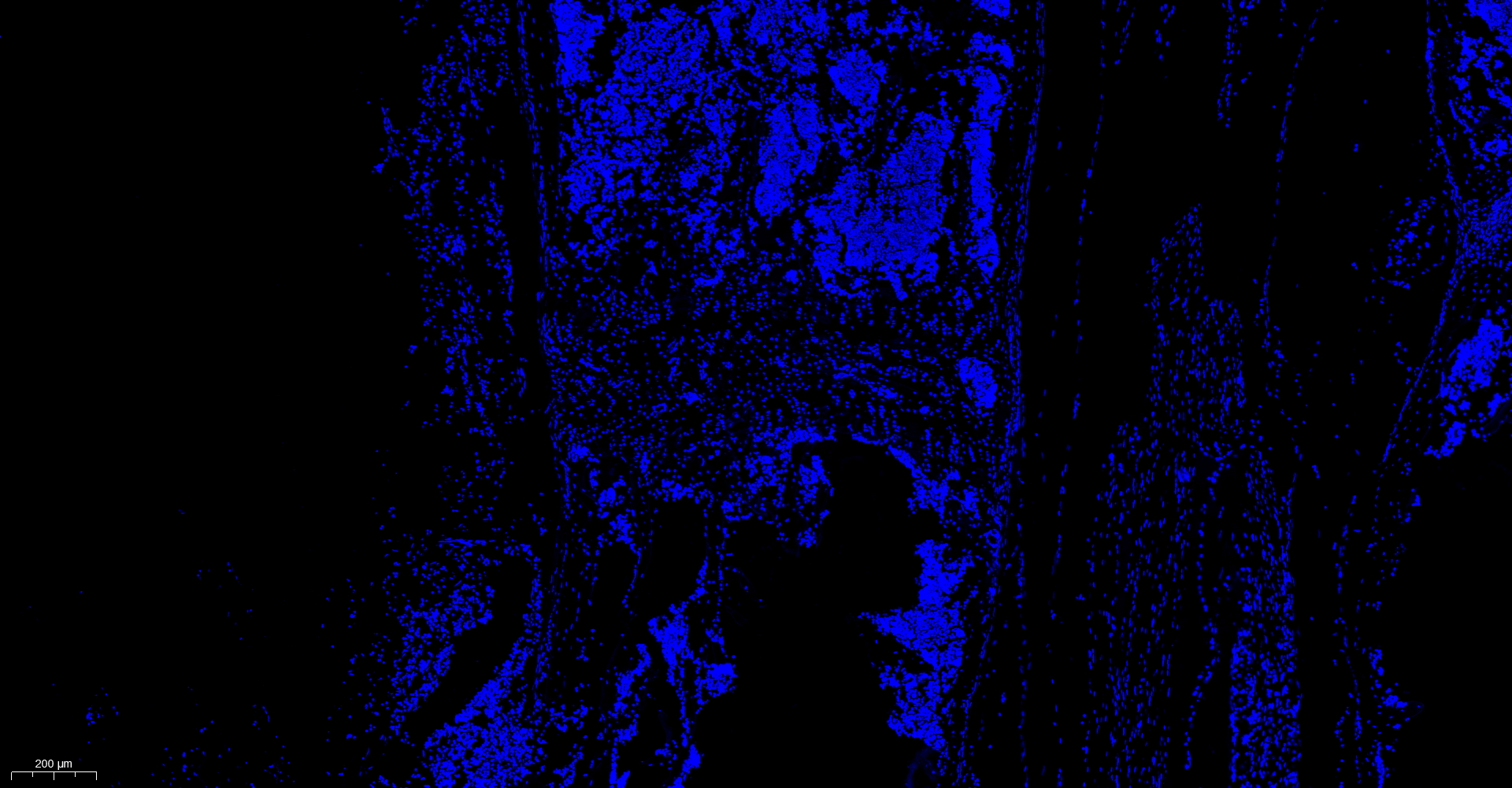

Supplement: Supplemental Information 4 [file peerj-14-20534-s004.zip › raw data4/Figure 4 D5.jpg]

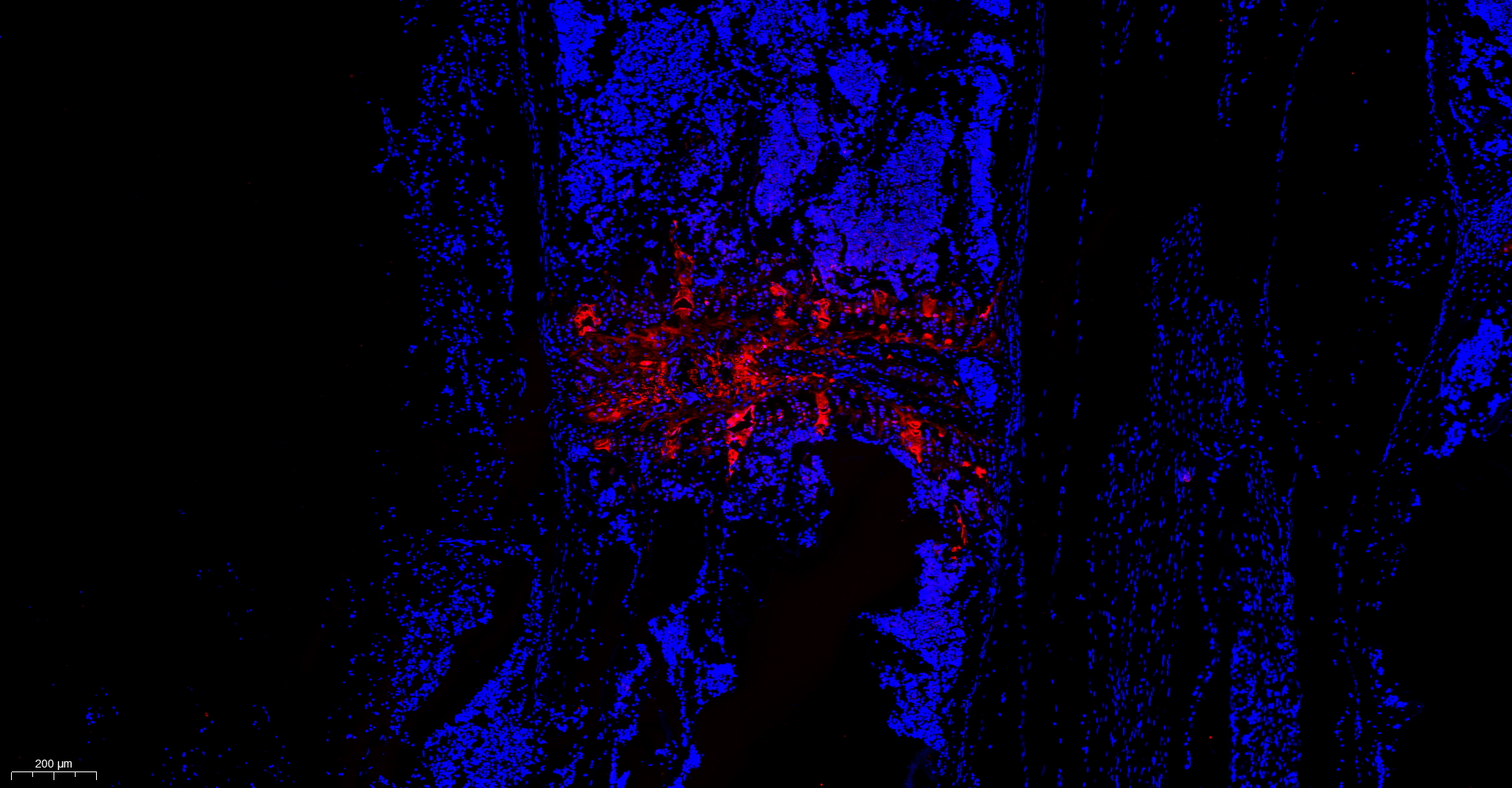

Supplement: Supplemental Information 4 [file peerj-14-20534-s004.zip › raw data4/Figure 4 D6.jpg]

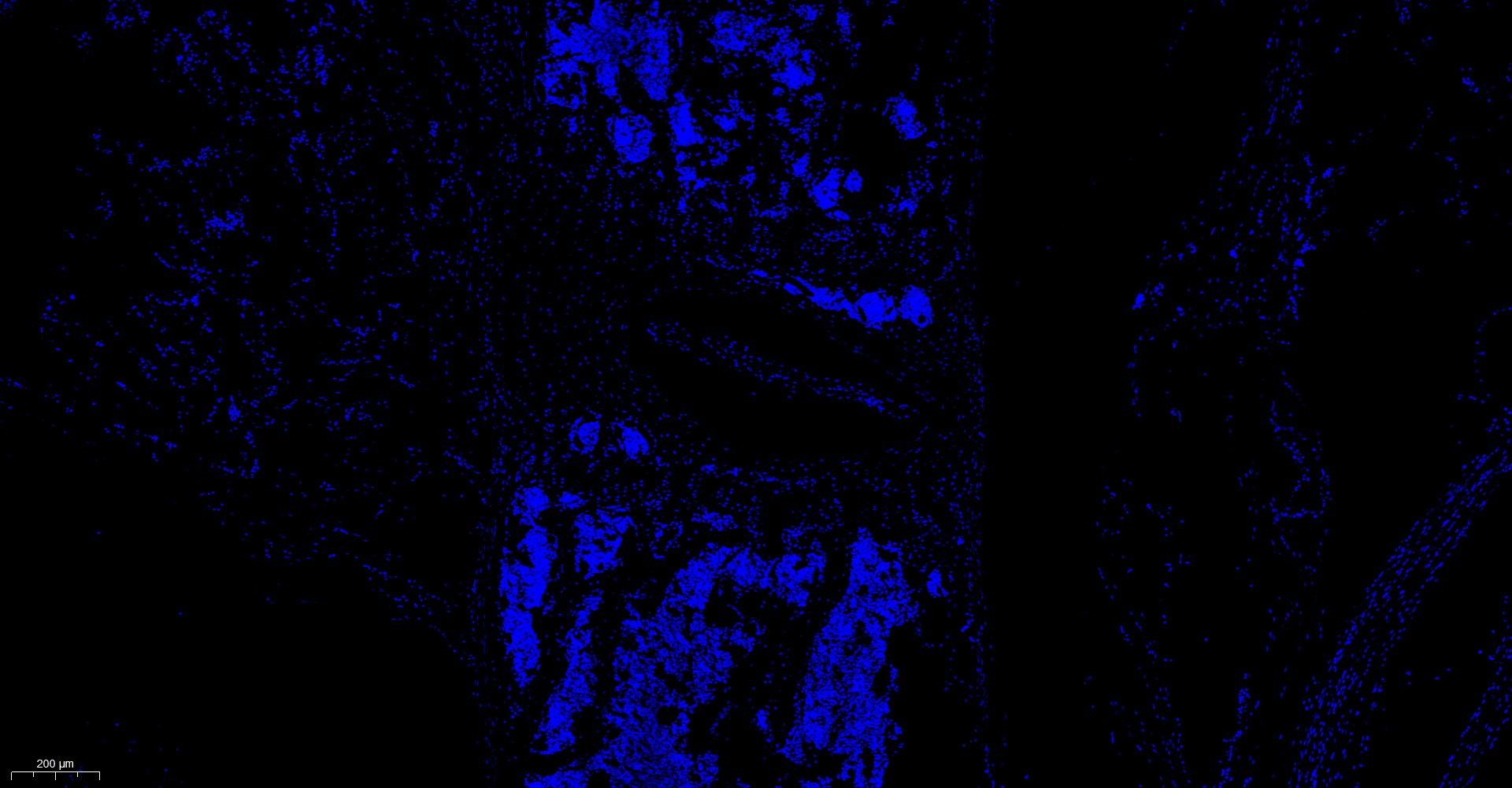

Supplement: Supplemental Information 4 [file peerj-14-20534-s004.zip › raw data4/Figure 4 E1.jpg]

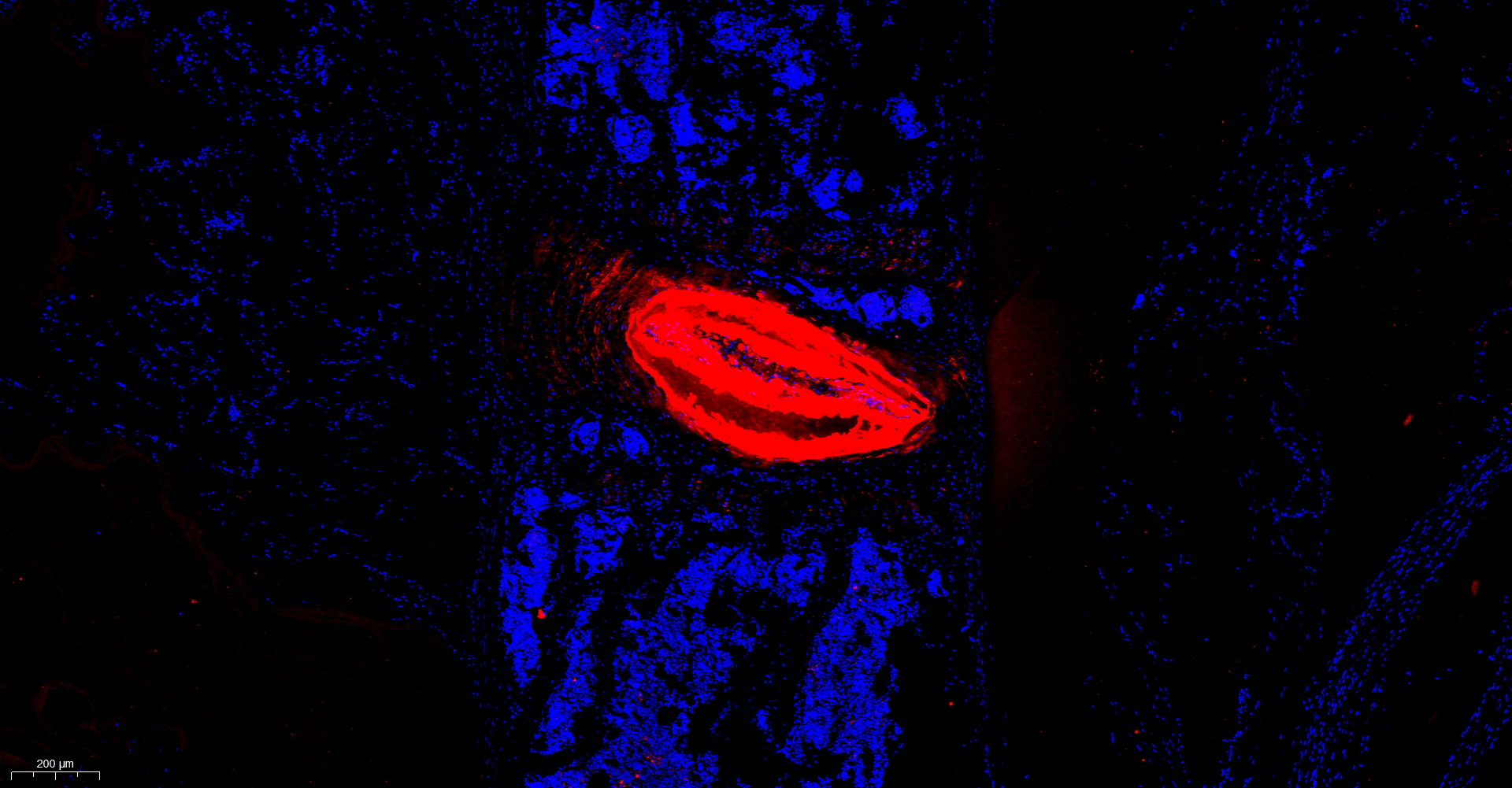

Supplement: Supplemental Information 4 [file peerj-14-20534-s004.zip › raw data4/Figure 4 E2.jpg]

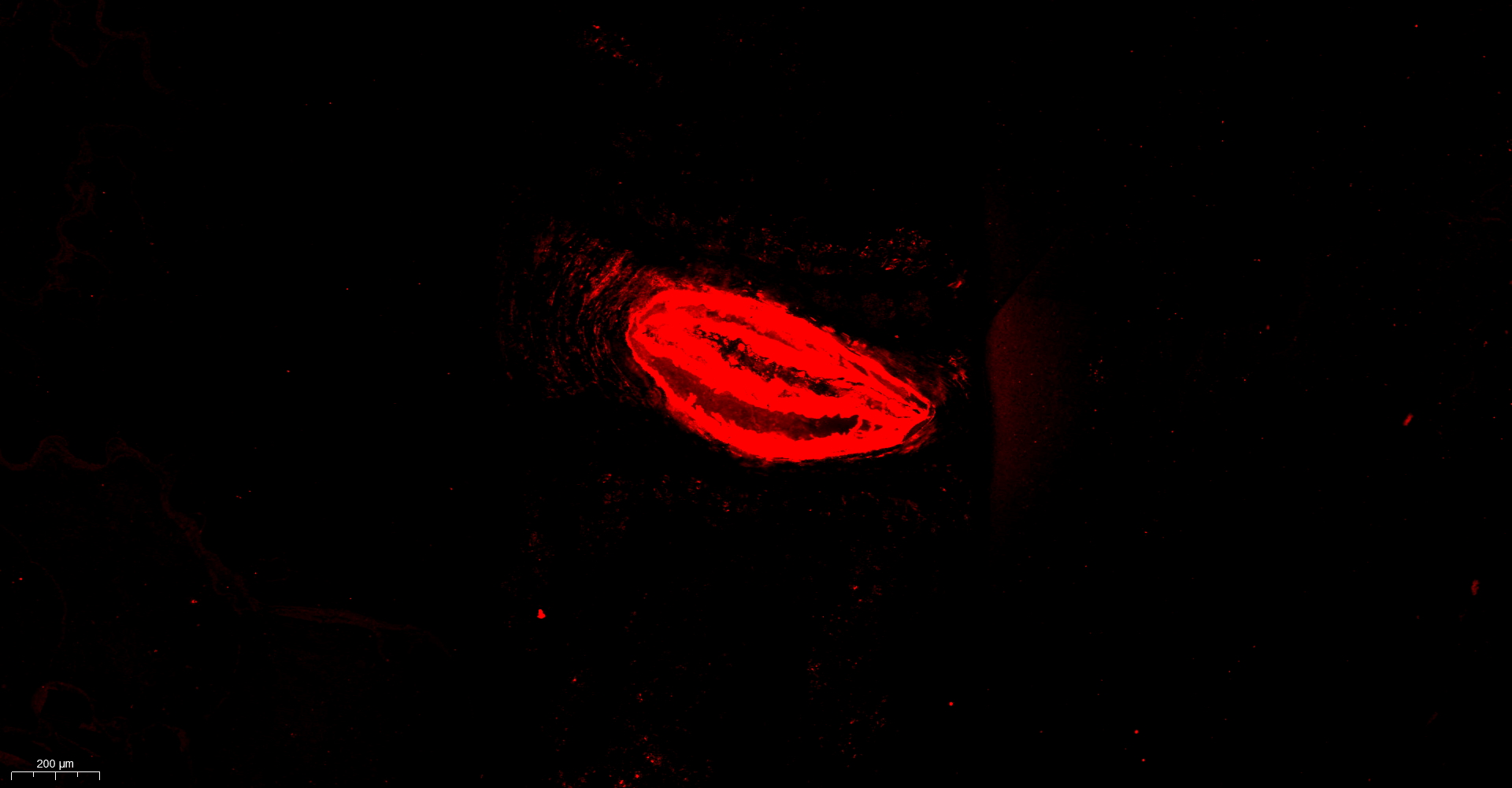

Supplement: Supplemental Information 4 [file peerj-14-20534-s004.zip › raw data4/Figure 4 E3.jpg]

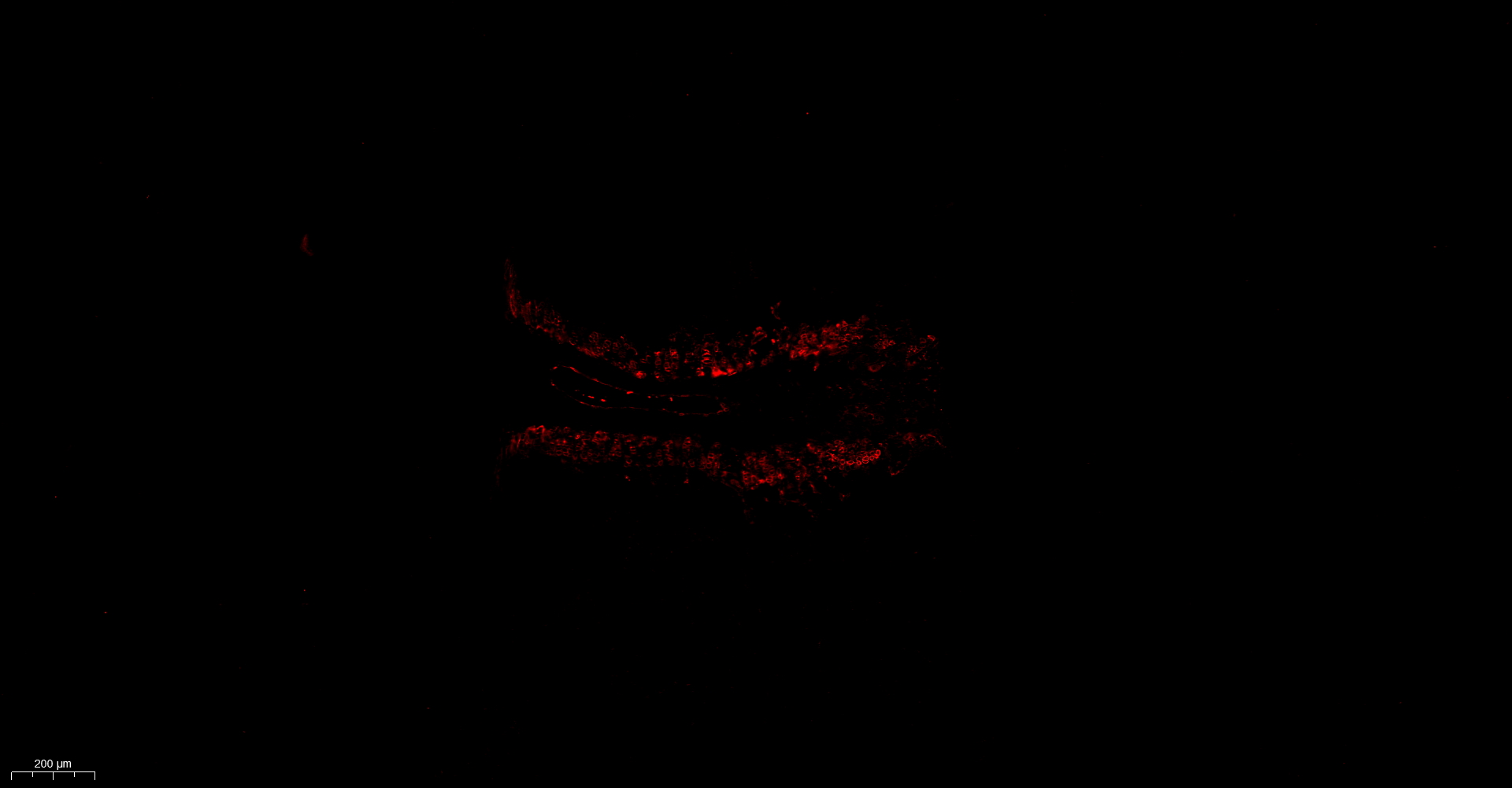

Supplement: Supplemental Information 4 [file peerj-14-20534-s004.zip › raw data4/Figure 4 E4.jpg]

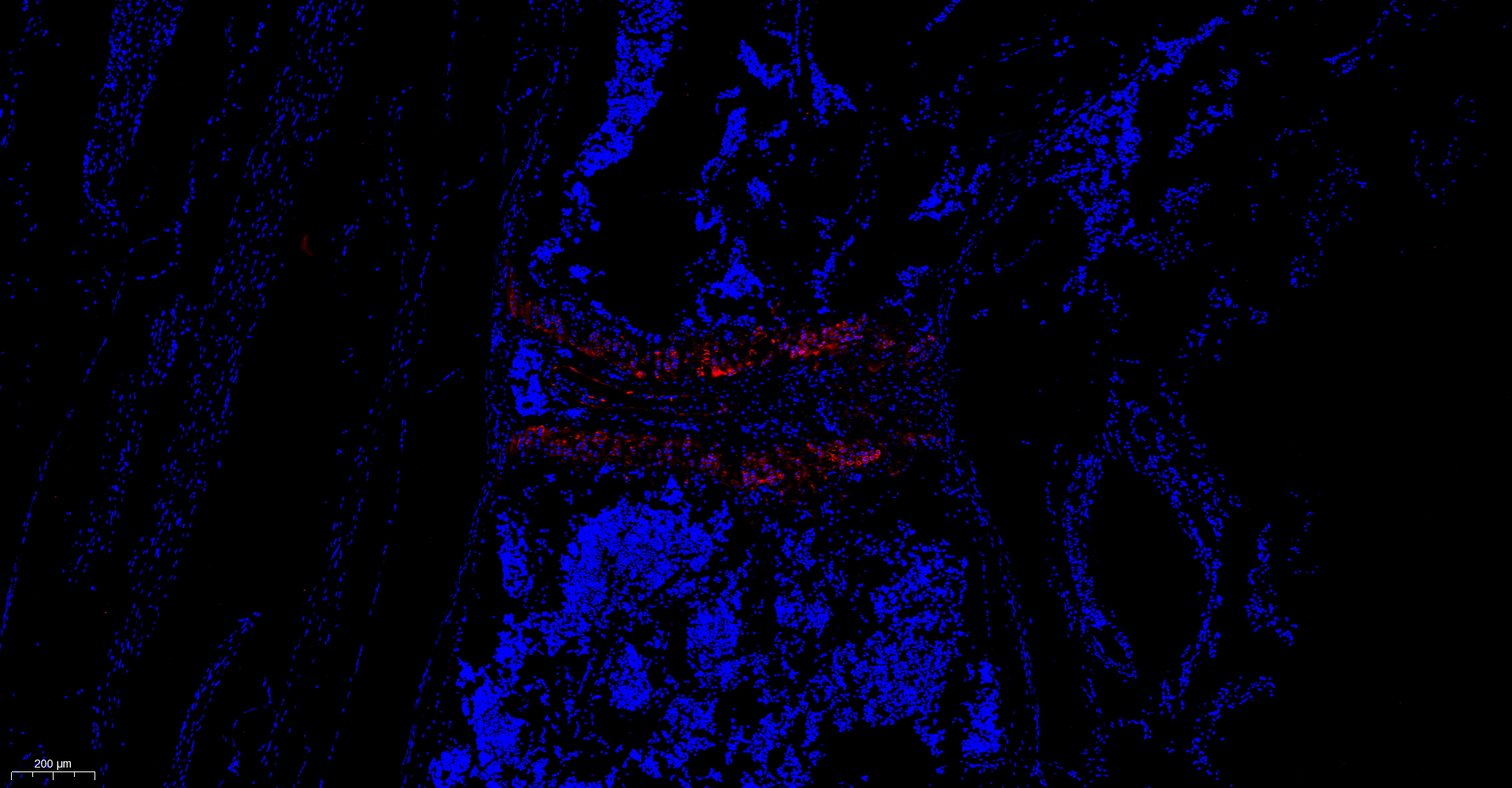

Supplement: Supplemental Information 4 [file peerj-14-20534-s004.zip › raw data4/Figure 4 E5.jpg]

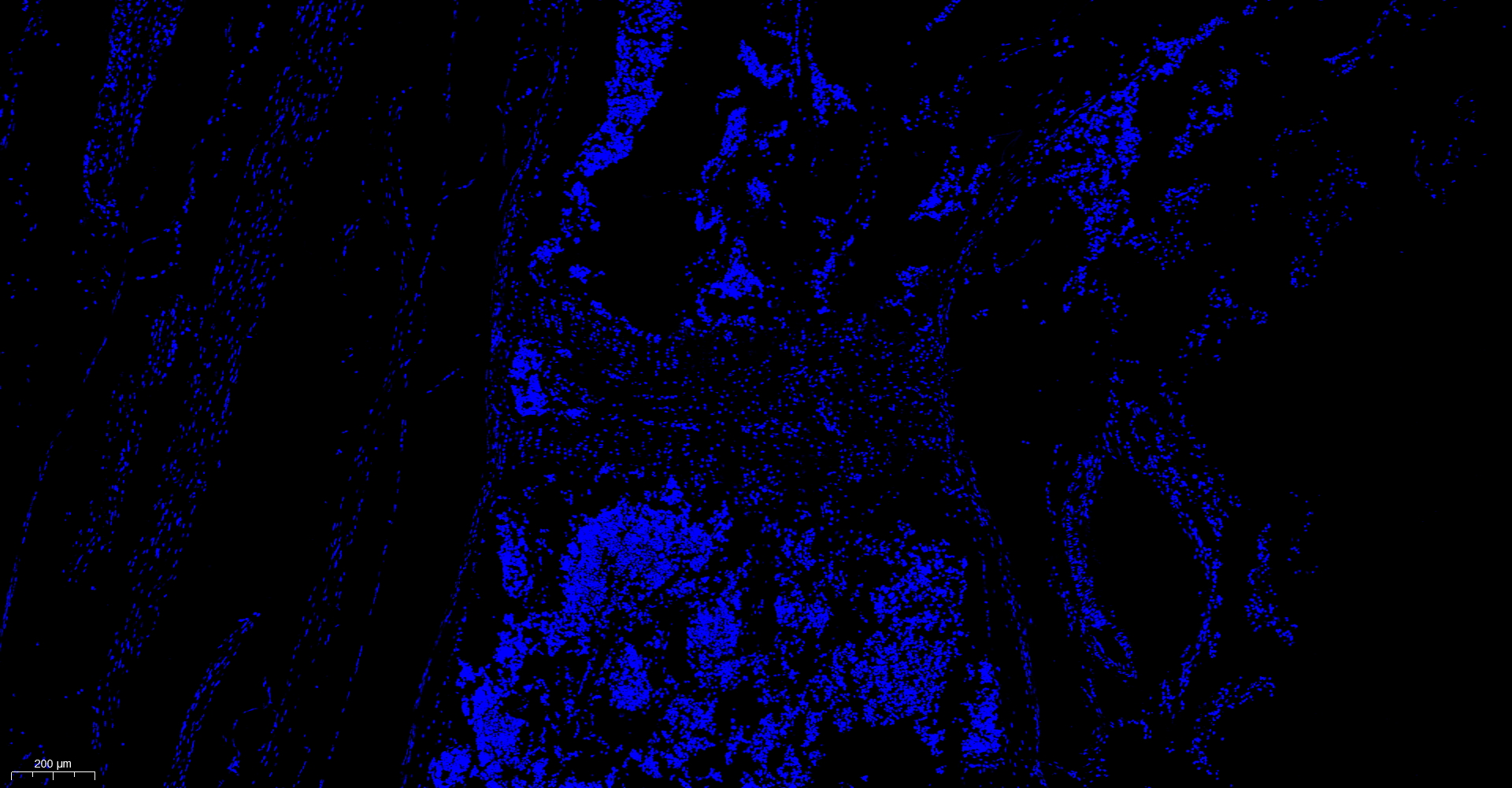

Supplement: Supplemental Information 4 [file peerj-14-20534-s004.zip › raw data4/Figure 4 E6.jpg]

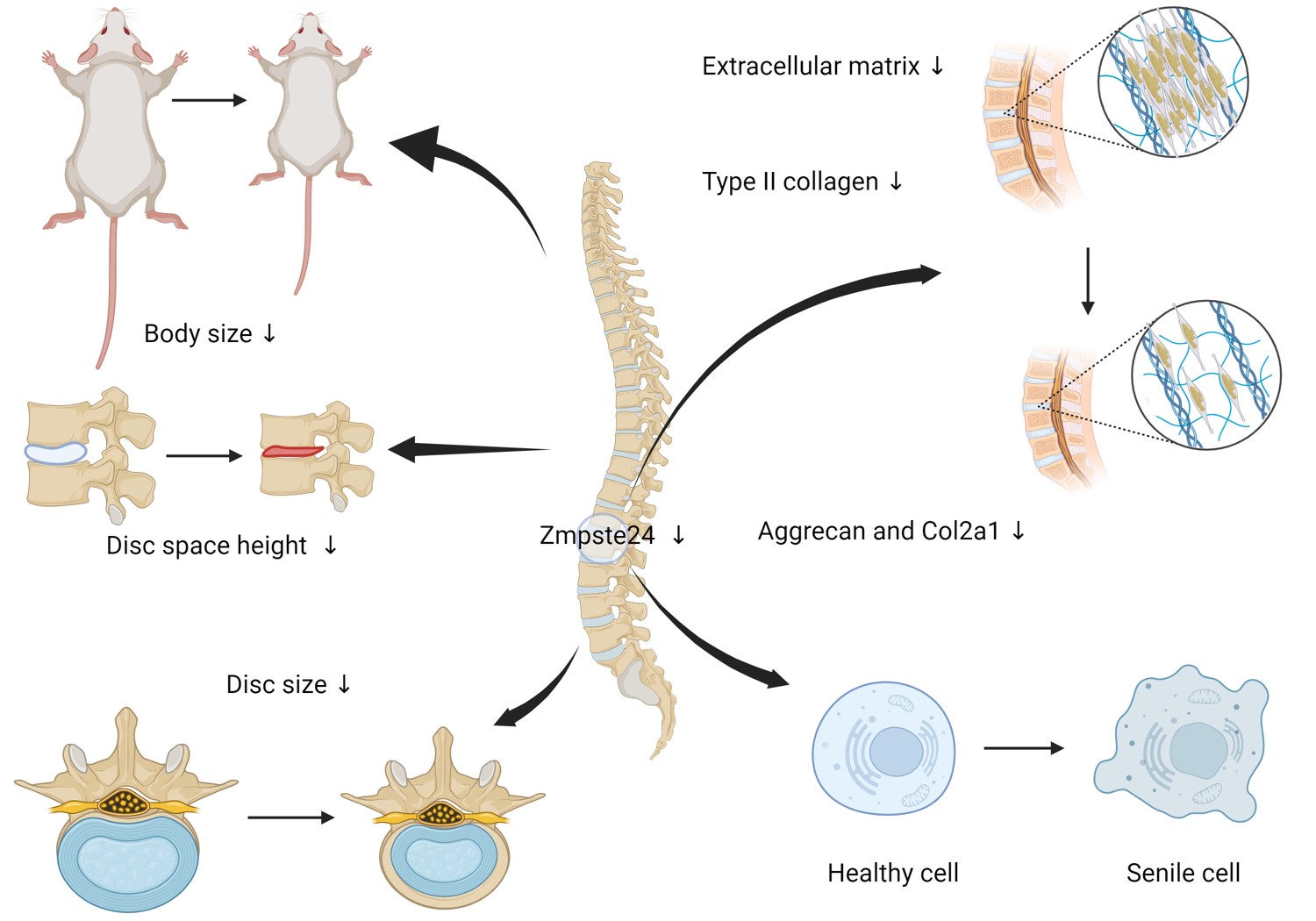

Supplement: Supplemental Information 4 [file peerj-14-20534-s004.zip › raw data4/GraphicalAbstract1.jpg]

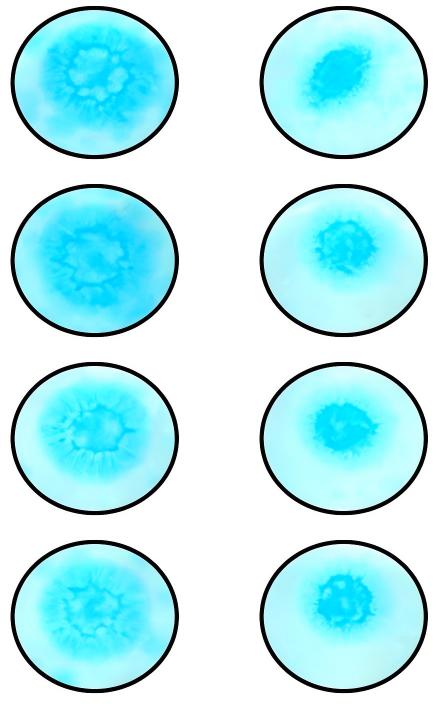

Supplement: Supplemental Information 6 [file peerj-14-20534-s006.zip › new supplementary Figures 2/Comparison of high-density culture area of rat NP cells.jpg]

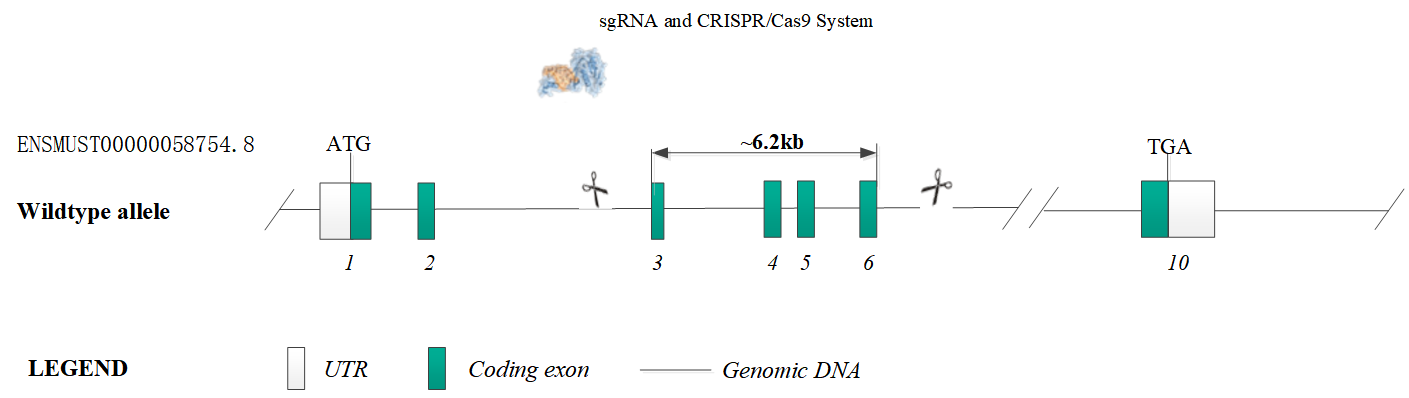

Supplement: Supplemental Information 6 [file peerj-14-20534-s006.zip › new supplementary Figures 2/Construction strategy of Zmpste24 KO mice.png]

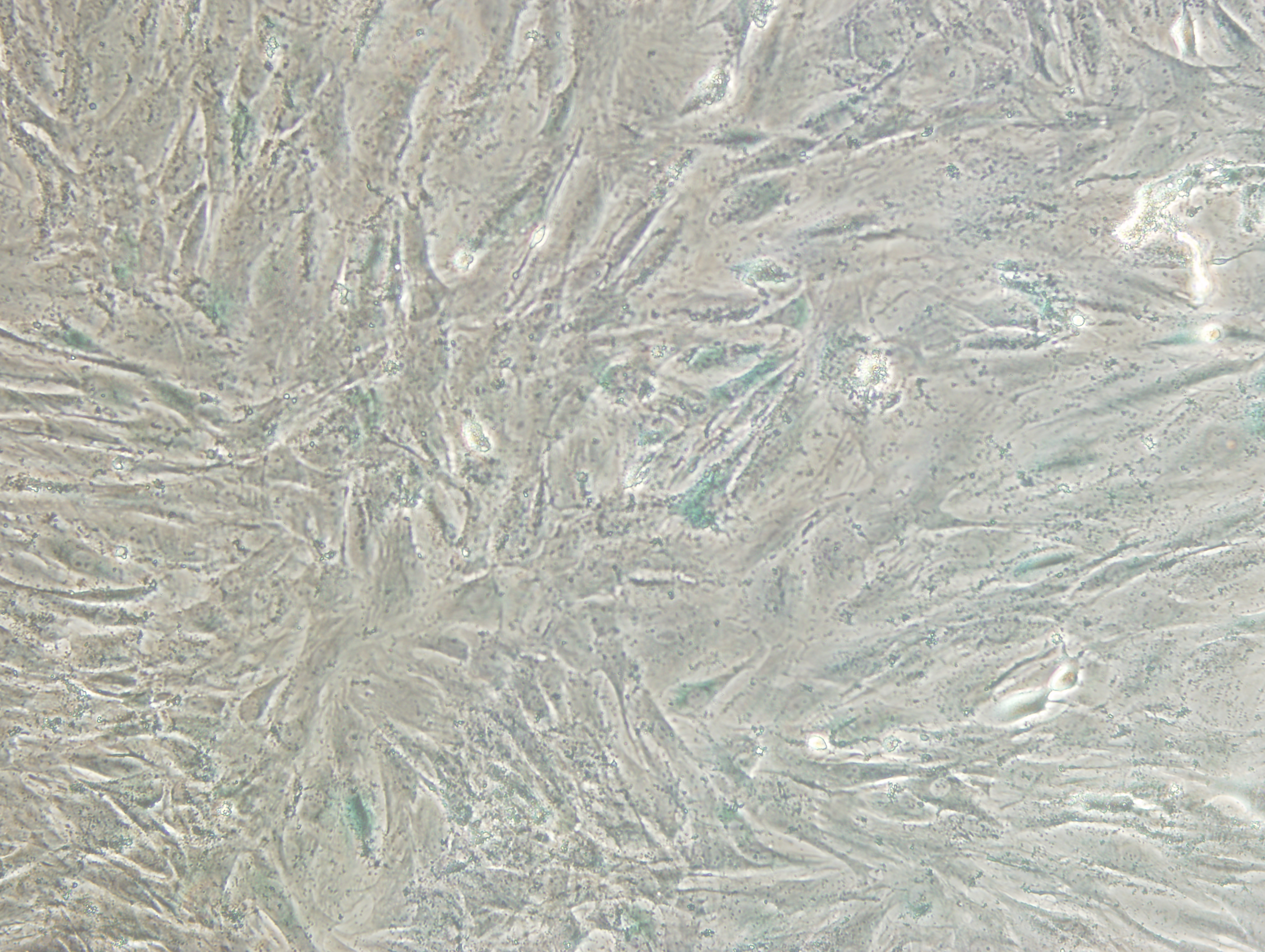

Supplement: Supplemental Information 6 [file peerj-14-20534-s006.zip › new supplementary Figures 2/β-gal Control.tif]
