# Supplementary material for: Zmpste24 deficiency contributes to intervertebral disc degeneration by undermining the stability of the nuclear membrane of nucleus pulposus cells
Source: PeerJ. 2026 Jan 7;14:e20534. doi: 10.7717/peerj.20534 (PMC12790285; doi:10.7717/peerj.20534)
Supplement: Supplemental Information 7 [file peerj-14-20534-s007.docx]

Figure 1 B：

| 3-4 | 5-6 | 7-8 |
| --- | --- | --- |
| 178.7286 | 56.80645 | 27.85714 |
| 171.4667 | 55.3125 | 27.59649 |
| 158.9375 | 54.81818 | 27.36207 |

Mean density of positive staining（IOD/cell number）

Figure 2 B

| Control | Lopinavir |
| --- | --- |
| 1.039076801 | 0.328213291 |
| 0.991961799 | 0.325984879 |
| 0.904098687 | 0.329805014 |
| 1.064862714 | 0.347313967 |

Comparison of high-density culture area of rat nucleus pulposus cells between contorl group and Lopinavir group

Figure 2 C

| control | Lopinavir |
| --- | --- |
| 0.955132534 | 0.806206245 |
| 1.044867466 | 0.804266576 |
| 1 | 0.80245 |

Relative RNA expression of Zmpste24

Figure 2 D

| control | Lopinavir |
| --- | --- |
| 1.008718965 | 0.835192126 |
| 1.000108857 | 0.797722974 |
| 0.991248494 | 0.881041986 |

Relative RNA expression of Col2a1

Figure 2 E

| control | Lopinavir |
| --- | --- |
| 1.061160776 | 0.829010845 |
| 1.016824168 | 0.891458833 |
| 0.922015056 | 0.892679099 |

Relative RNA expression of Lamin A/C

Figure 2 F

| control | Lopinavir |
| --- | --- |
| 0.992044726 | 1.184371395 |
| 0.946657834 | 1.321756374 |
| 1.064818809 | 1.189362775 |

Relative RNA expression of Mmp3

Figure 3 E

| WT♂ | Zmpste24 KO♂ | WT♀ | Zmpste24 KO♀ |
| --- | --- | --- | --- |
| 104 | 53 | 93 | 50 |
| 102 | 58 | 96 | 48 |
| 94 | 54 | 92 | 53 |

Statistical table of DHI% of the four mouse species

Figure 4 B

| WT | *Zmpste24* hets | *Zmpste24* KO |
| --- | --- | --- |
| 1 | 4 | 5 |
| 1 | 3 | 6 |
| 1 | 3 | 6 |

Statistical table of histological scale in mice of the three genotypes

Figure 4 F

| WT | *Zmpste24* KO |
| --- | --- |
| 1.036644458 | 0.166717141 |
| 0.94488189 | 0.149182314 |
| 1.018473652 | 0.156359782 |

Statistical table of the relative fluorescence intensity of COL2A1

Figure 4 G

| WT | *Zmpste24* KO |
| --- | --- |
| 1.001165501 | 0.108974359 |
| 1.006993007 | 0.119347319 |
| 0.991841492 | 0.116317016 |

statistical table of the relative fluorescence intensity of AGGRECAN

**Supplementary Table 1** the sgRNA sequence for *Zmpste24* knockout

| sgRNA | 5’→ 3’ | PAM |
| --- | --- | --- |
| 5S1 | AAGGCTACGTAGTGAGCTCT | TGG |
| 3S1 | CAGGGCATAAGCATGGAACC | TGG |

**Supplementary Table 2** Primer Sequences

| **Gene** | **Forward Primer (5′→3′)** | **Reverse Primer (5′→3′)** |
| --- | --- | --- |
| *β-actin* | CCCGCGAGTACAACCTTCT | ATGCCGTGTTCAATGGGGTA |
| *Zmpste24* | GTGGACGCTATGTGGGACTT | TCTGGTGGTACATGAGTCGT |
| *Mmp3* | CCTCTGAGTCTTTTCATGGAGGG | ACTTGAGGTTGACTGGTGCC |
| *LMNA* | CGCAAGCTGGAGTCTTCTGA | CTCCTGAAGCCCAGATCGTC |
| *Col2a1* | GCCAGGATGCCCGAAAATTAG | GGCTGCAAAGTTTCCTCCAC |
| *p16* | CGAACTCGAGGAGAGCCATC | GGGGTACGACCGAAAGAGTT |
| *p21* | TAAGGACGTCCCACTTTGC | GACAACGGCACACTTTGCTC |
